# Multiframe Evolving Dynamic Functional Connectivity *(EVOdFNC)*: A Method for Constructing and Investigating Functional Brain Motifs

**DOI:** 10.3389/fnins.2022.770468

**Published:** 2022-04-19

**Authors:** Robyn L. Miller, Victor M. Vergara, Godfrey D. Pearlson, Vince D. Calhoun

**Affiliations:** ^1^The Tri-Institutional Center for Translational Research in Neuroimaging and Data Science (TReNDS): Georgia State University, Georgia Institute of Technology and Emory University, Atlanta, GA, United States; ^2^Yale School of Medicine, New Haven, CT, United States

**Keywords:** functional magnetic resonance imaging (fMRI), functional network connectivity (FNC), dynamic functional network connectivity (dFNC), schizophrenia, resting state fMRI, uniform manifold approximation and embedding (UMAP)

## Abstract

The study of brain network connectivity as a time-varying property began relatively recently and, to date, has remained primarily concerned with capturing a handful of discrete static states that characterize connectivity as measured on a timescale shorter than that of the full scan. Capturing group-level representations of temporally evolving patterns of connectivity is a challenging and important next step in fully leveraging the information available in large resting state functional magnetic resonance imaging (rs-fMRI) studies. We introduce a flexible, extensible data-driven framework for the stable identification of group-level multiframe (movie-style) dynamic functional network connectivity (dFNC) states. Our approach employs uniform manifold approximation and embedding (UMAP) to produce a continuity-preserving planar embedding of high-dimensional time-varying measurements of whole-brain functional network connectivity. Planar linear exemplars summarizing dominant dynamic trends across the population are computed from local linear approximations to the two-dimensional 2D embedded trajectories. A high-dimensional representation of each 2D exemplar segment is obtained by averaging the dFNC observations corresponding to the n planar nearest neighbors of τ evenly spaced points along the 2D line segment representation (where n is the UMAP number-of-neighbors parameter and τ is the temporal duration of trajectory segments being approximated). Each of the 2D exemplars thus “lifts” to a multiframe high-dimensional dFNC trajectory of length τ. The collection of high-dimensional temporally evolving dFNC representations (EVOdFNCs) derived in this manner are employed as dynamic basis objects with which to characterize observed high-dimensional dFNC trajectories, which are then expressed as weighted combination of these basis objects. Our approach yields new insights into anomalous patterns of fluidly varying whole-brain connectivity that are significantly associated with schizophrenia as a broad diagnosis as well as with certain symptoms of this serious disorder. Importantly, we show that relative to conventional hidden Markov modeling with single-frame unvarying dFNC summary states, EVOdFNCs are more sensitive to positive symptoms of schizophrenia including hallucinations and delusions, suggesting that a more dynamic characterization is needed to help illuminate such a complex brain disorder.

## Introduction

The investigation of functional brain network connectivity (FNC) as a time-varying property in resting state functional magnetic resonance imaging (rs-fMRI) studies began relatively recently and, to date, has remained primarily concerned with capturing a handful of discrete static states that characterize connectivity as measured on a timescale shorter than that of the full scan (Allen et al., 2014; Damaraju et al., 2014; Ou et al., 2015; Yaesoubi et al., 2015, 2017; Abrol et al., 2017a,b; Marusak et al., 2017; Barber et al., 2018; Diez-Cirarda et al., 2018; Faghiri et al., 2018; Patanaik et al., 2018; Rashid et al., 2018; Smith et al., 2018; Vergara et al., 2018; Xie et al., 2018, 2019; Denkova et al., 2019; Espinoza et al., 2019a; Fiorenzato et al., 2019; Fu et al., 2019; Gonzalez-Castillo et al., 2019; Hou et al., 2019; Klugah-Brown et al., 2019; Li et al., 2019, 2021; Liu et al., 2019; Mash et al., 2019; Rabany et al., 2019; Yao et al., 2019; Zhou et al., 2019; Agcaoglu et al., 2020; d’Ambrosio et al., 2020; Mennigen et al., 2020; Shappell et al., 2021). Temporal variation in fMRI has been employed primarily to establish evidence of stable hemodynamic covariation between pairs of functionally or anatomically defined brain regions or functionally coherent distributed spatial networks. Although initially controversial (Laumann et al., 2017; Liegeois et al., 2017; Miller et al., 2018; Lurie et al., 2020), research extending this paradigm from so-called “static” scan-length patterns of functional integration into the analysis of transient but replicable patterns of covariation between functional networks has gained a strong foothold in recent years (Calhoun et al., 2014; Zalesky et al., 2014; Breakspear, 2017; Keilholz et al., 2017; Preti et al., 2017; Heitmann and Breakspear, 2018; Khambhati et al., 2018). A substantial amount of this work, however, focuses on the separation of windowed, time-resolved connectivity measures into temporally static patterns that are consistently transiently realized across subjects. Typically, the dynamics are then treated as a discrete (memoryless) Markov process, characterized by the probability of transitioning from any one of the summary states at time *t* to the same or another at time *t + 1*. The simplifying assumptions that (1) a small number of snapshot summary connectivity patterns capture the functionally important variations large-scale brain connectivity on shorter timescales and that, furthermore, and (2) brain dynamics are Markovian are useful starting points but stop short of revealing how complex, fluidly varying reconfigurations of whole-brain connectivity reflect the myriad dimensions of brain health and dysfunction researchers seek to understand. Capturing group-level representations of temporally evolving patterns of connectivity is a challenging and important next step in fully leveraging the information available in large rs-fMRI studies. We introduce a flexible, extensible data-driven framework for the identification of group-level multiframe (movie-style) dynamic functional network connectivity (dFNC) states. Our approach employs uniform manifold approximation and embedding (UMAP) to produce a planar embedding of the high-dimensional whole-brain connectivity dynamics that preserves important characteristics, such as trajectory continuity, of the dynamics in the native high-dimensional state space. The method is shown to produce naturalistic (i.e., smoothly spatiotemporally varying through sequences of realistic connectomic patterns), interpretable, and evolving dFNC motifs (EVOdFNCs) whose role in the dynamic connectomes of schizophrenia patients (SZs) and healthy controls (HCs) differs significantly and interpretably. Furthermore, these evolving multiframe representations of dynamic connectivity exhibit stronger relationships with specific positive symptom scores in patients than traditional static representations of time-resolved connectivity, suggesting that methods such as ours hold promise for extracting more effective dynamical biomarkers from rs-fMRI than have thus far been possible.

**FIGURE 1:**
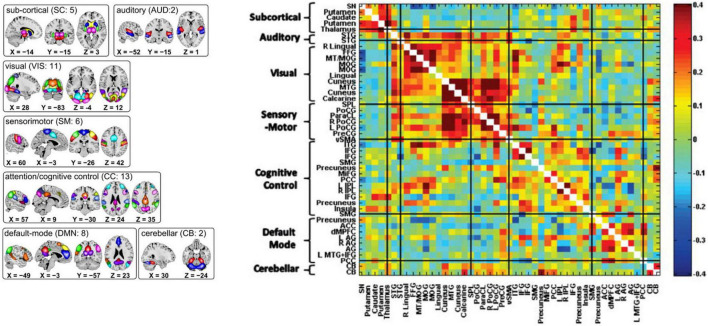
(Left) Composite maps of the 47 resting state networks use in this study, organized according to functional domain with each network in the indicated domain shown in a different color (Damaraju et al., 2014); (Right) Population means of pairwise correlations between RSN time series. The order in which networks and functional domains (shown along the *y*-axis) are presented here is consistent through all figures in this paper. The functional domains, indicated by bracketed groups of networks on the *y*-axis, are most often displayed in abbreviated form: SC, subcortical; AUD, auditory; VIS, visual; SM, sensorimotor; CC, cognitive control; DMN, default mode network; and CB, cerebellar.

**FIGURE 2:**
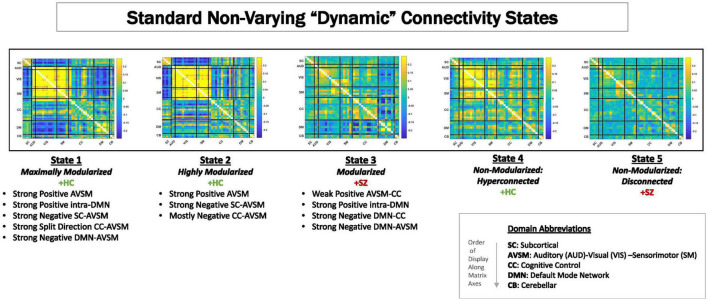
Centroids from clustering time-resolved connectivity measures without in a time-blind manner. These are called “dFNC states.” For these data, as have been shown in previous publications (Damaraju et al., 2014; Miller et al., 2016c; Yaesoubi et al., 2017; Espinoza et al., 2019a; Rashid et al., 2019), there are five characteristic states that can be can be visually summarized as ranging from maximally modularized to weakly connected. Studies consistently show that schizophrenia patients spend more time in States 3 and 5 on average than do controls, whereas controls spend more time on average than patients in States 1, 2, and 4.

**FIGURE 3:**
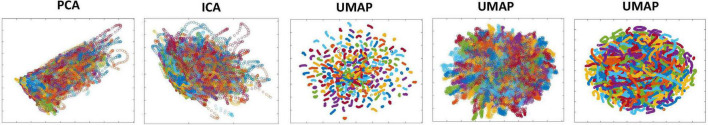
Applying different data reduction methods to the 1,081-dimensional dFNC observations yields various planar presentations. PCA (column 1) and spatial Independent Component Analysis (ICA) (column 2) are “cloudy” and do not produce continuous, well-defined, subject-level trajectories; UMAP with n_neighbors = 200 and min_dist = 0.1 (column 3) has a large periphery of isolated geometrically compressed trajectories; UMAP with n_neighbors = 200 and min_dist = 0.8 (column 4) results in a crowded but diffuse embedding that blurs within-subject temporal trajectory continuity. UMAP with the parameters used in this study, n_neighbors = 25 and min_dist = 0.75, embeds individual subject trajectories as continuous curves that are densely intermingled at a group level, qualitatively reflecting the high-dimensional reality. In all panels, points corresponding to each subject are plotted in the same color.

**FIGURE 4:**
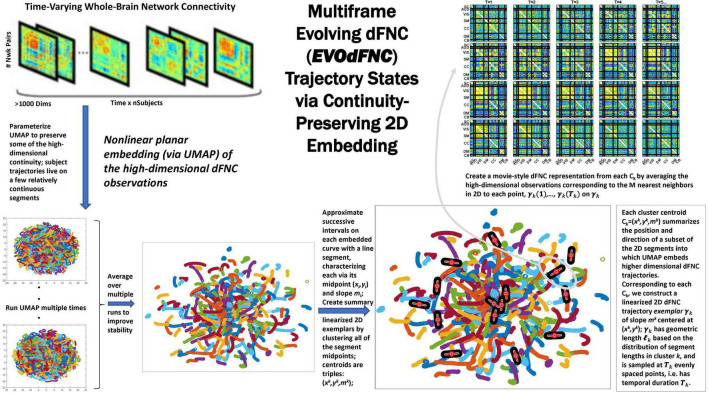
Overview schematic exhibiting the pipeline for producing representative evolving multiframe high-dimensional states of dynamic functional network connectivity (EVOdFNCs). High-dimensional dynamic functional connectivity assessed as pairwise correlations on sliding windows through each subject’s GICA network timecourses (top left) are embedded into 2D using uniform manifold approximation and projection (UMAP). The embedding is done *R* times (bottom leftmost), then averaged over the *R* runs to improve stability (bottom second from the left). Continuous trajectories in the averaged embedding are treated as locally (i.e., sub-segments of temporal duration τ = 44) linear. Directional and positional trends in the 2D dynamics are then captured by clustering the midpoints and slopes of these local linearizations, resulting in a set of summary tangents to the embedded trajectories, linearized trajectory exemplars (bottom right). The exemplars are then “inverted” back to the native dFNC dimensionality, as EVOdFNCs, by associating to each of τ evenly points along an exemplar, the average of the high-dimensional dFNCs that map to that point’s *n* nearest neighbors in 2D (top right).

**FIGURE 5:**
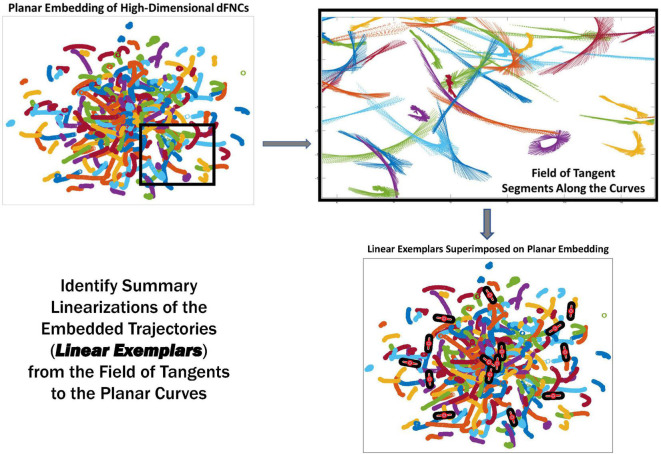
The linear trajectory segments (LTSs) (top right) along the boxed embedded trajectories (top left). These local linearizations are clustered to identify a set of linear trajectory exemplars (bottom right, superimposed black and red segments) capturing localized directional trends in the dynamics.

**FIGURE 6:**
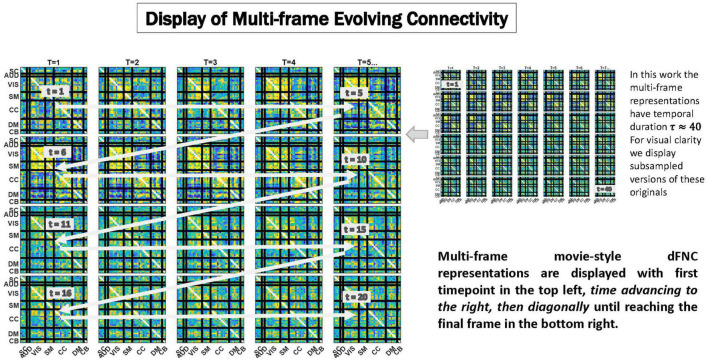
We display temporally evolving dFNCs as a sequence of frames in which the first time point is always the top leftmost subplot, and with time advancing from left to right, then clockwise diagonally back to the first subplot in the subsequent row, until reaching the final frame of the sequence shown in the bottom rightmost subplot. Because these are very dense displays, the EVOdFNCs in this work, which have duration 44 time points are subsampled to show only every other frame in figures below. To avoid more crowding, colorbars are not displayed, but the range is fixed across EVOdFNCs, centered at 0 and bounded in [−*q*, *q*] where *q* denotes the 95*^th^* percentile of all magnitudes in the collection of EVOdFNCs.

**FIGURE 7:**
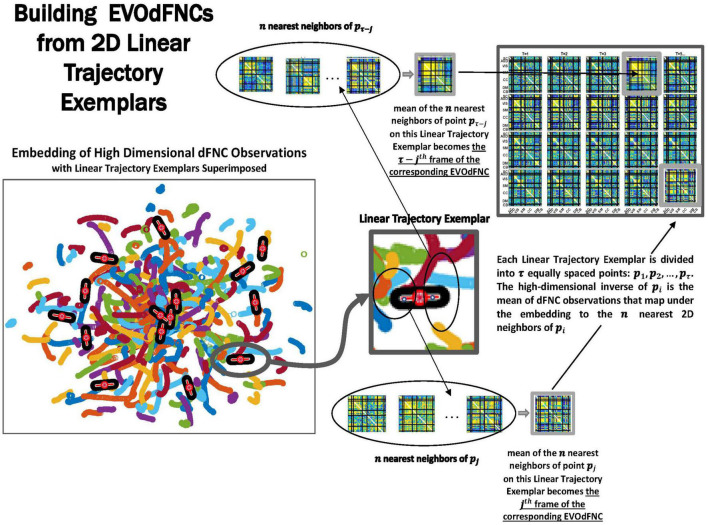
Exemplars are divided into τ evenly spaced points: *p*_1_, *p*_2_, … *p*_τ_ (where τ is the mean temporal duration of the piecewise linear approximations of continuous trajectories that are inputs to the clustering). The geometric length of each exemplar is the based on the lengths of the continuous trajectory segments belonging to the cluster they represent. Timepoint *t* = *i*, *i* = 1, 2, … , τ of the multifrane EVOdFNC corresponding to linear exemplar *k* is the average of the observed high-dimensional dFNCs corresponding to the *n* = 25 nearest 2D neighbors of *p*_*i*_.

**FIGURE 8:**
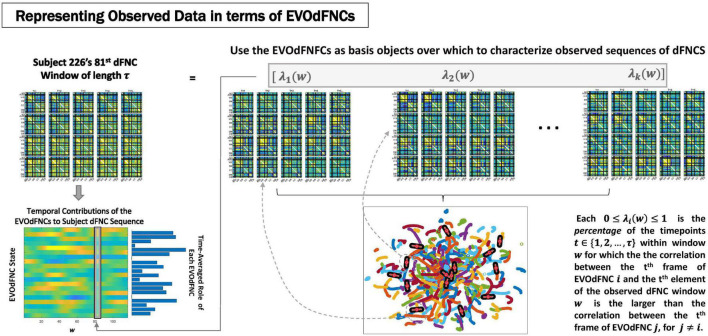
Represent each length-τ window w from the observed subject dFNCs as a weighted combinations of the K EVOdFNC states (top row); Rather than simply regressing full 1081τ = 47,564-dimensional dFNC windows on the *K*1081τ-dimensional EVOdFNCs, which ends up highlighting the relationship, we go time point–wise through the data window and EVOdFNCs, expressing each time point *t* of the data in terms of the corresponding time point in each EVOdFNC, then averaging this evolving correspondence over the window *w* between the observation and each EVOdFNC *k* to obtain the weight β_*k*_(*w*) for the *k^th^* EVOdFNC on the *r^th^* length-τ window of the subject’s dFNC sequence. This yields a *K*-variate time series expressing the subject’s observed sequence of dFNCs in terms of time-indexed weightings on the EVOdFNCs (bottom left).

**FIGURE 9:**
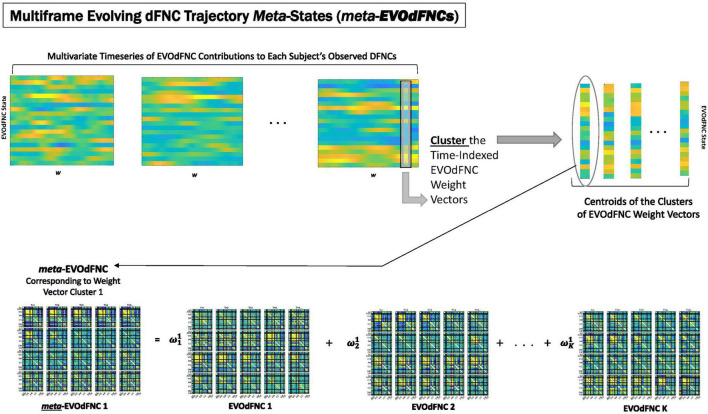
EVOdFNCs are high-dimensional data-driven inverses of exemplar 2D linear trajectories that are relatively sparse in the space of linearized 2D trajectory segments. A fuller range of the structure and dynamics exhibited by actual high-dimensional dFNC observations can be captured by expressing each length-τ observed dFNC sequence as a weighted combination of the EVOdFNCs. From the multivariate EVOdFNC time series we induce a collection of meta-EVOdFNCs by clustering the time-indexed weight vectors (top row) and applying the weight-vector cluster centroids to the EVOdFNC basis states (bottom row).

Because of its complexity, we present the methodological pipeline in a series of schematics covering different stages at different levels of granularity. For ease of navigation, we will lead off with a short guided tour of Materials and Methods section figures and schematics: Figure 1 displays the underlying networks (Damaraju et al., 2014) and functional domains (Miller et al., 2016a) in the order they appear in all future figures featuring functional network connectivity plots. It also shows the average static connectivity between these networks. In this figure, the network and domain labels along the axes are displayed in a larger font than they are in some of the denser figures and are easier to read. Figure 2 is also included for background. It displays the so-called “dynamic states” obtained by clustering time-resolved functional network connectivity computed on sliding windows through the multivariate network time courses (TCs), along with short descriptive summaries and schizophrenia effects on the state occupancy rates. These states and clinical findings were first reported in Damaraju et al. (2014) and, afterward, appeared in other published work, e.g., (Miller et al., 2016b,c, 2018; Espinoza et al., 2019b; Rashid et al., 2019). Figure 3 contrasts several alternative approaches to planar embedding with the one chosen (shown in the far right panel in the figure). Figure 4 provides a comprehensive schematic overview of all steps in the pipeline, from the initial computation of windowed network connectivity, through the embeddings, the averaging process, the computation of local linear approximations, and the extraction of planar linear exemplars, ultimately lifting these exemplars into higher-dimensional EVOdFNCs. Figure 5 puts the linearizations near embedded planar curves under magnification to elucidate the objects from which linear exemplars are distilled. Figure 6 fixes the format of display for the high-dimensional evolving (multiframe) movie-style connectivity motifs. Figure 7 is a highly detailed schematic focused on exactly how the planar linear exemplars are lifted into high-dimensional multiframe evolving connectivity motifs. Figure 8 illustrates how the representational importance (RI) of evolving connectivity motifs corresponding to each of the planar linear exemplars is computed for empirical sequences of windowed connectivity observations. Finally, Figure 9 schematically presents the process by which RI of evolving connectivity motifs can be used to induce empirically informed blended or “meta”-level representations of evolving high-dimensional connectivity.

## Materials and Methods

### Data

We use data from a large, multi-site eyes-open resting state fMRI study with approximately equal numbers of SZs and HCs (*n* = 311, nSZ = 150). Imaging data for six of the seven sites were collected on a 3T Siemens Tim Trio System and on a 3T General Electric Discovery MR750 scanner at one site. Resting state fMRI scans were acquired using a standard gradient-echo echo planar imaging paradigm: FOV of 220 × 220 mm (64 × 64 matrix), TR = 2 s, TE = 30 ms, FA = 770, 162 volumes, and 32 sequential ascending axial slices of 4 mm thickness and 1 mm skip. Subjects had their eyes closed during the resting state scan. The data were preprocessed with a standard pipeline and decomposed with group-independent component analysis (GICA) into 100 group-level functional network spatial maps (SMs) with corresponding subject-specific TCs. Through a combination of automated and manual pruning, *N = 47* functionally identifiable resting state networks (RSNs) were retained (Figure 1). The remaining network TCs were detrended, despiked, and orthogonalized with respect to estimated subject motion parameters. Subject-specific SMs and TCs were obtained from the group level SMs *via* spatiotemporal regression. The TCs were detrended, despiked, and subjected to some additional post-processing steps [details in Damaraju et al. (2014)]. The retained RSNs fall into seven functional domains: subcortical (SC) (five networks), auditory (AUD) (two networks), visual (VIS) (10 networks), sensorimotor (SM) (seven networks), cognitive control (CC) (13 networks), default mode network (DMN) (eight networks), and cerebellum (CB) (two networks). In all figures showing functional network connectivity matrices in heatmap form, the functional domains appear in the indicated order along both axes. All subjects in the study signed informed consent forms. Symptom scores for patients were determined using the Positive and Negative Syndrome Scale (PANSS) (Kay et al., 1987). The focus in this paper will be on the six symptoms (delusions, grandiosity, hallucinations, suspiciousness/persecution, preoccupation, and unusual thought) that load on the “positive factor” in a heavily used factor analysis study of PANSS symptoms (Lindenmayer et al., 1995).

### Dynamic Functional Network Connectivity

Dynamic functional connectivity (dFNC) between RSN TCs was estimated using sliding window correlations. Following the protocols from published studies on dynamic connectivity (Damaraju et al., 2014), we employed a tapered rectangular window length of 22 TRs (44 s), advanced 1 TR at each step, and computed pairwise correlations between RSN TCs within these windows. After dropping the first three and final TRs, this procedure yields a 47(47−1)/2 = 1,081-dimensional dFNC measure on each of *136* windows of length *22*TRs for each subject. Clustering this collection of time-resolved connectivity observations using MATLAB’s implementation of k-means clustering (Euclidean distance, 2,000 iterations, 250 repetitions, five clusters chosen according the elbow criterion) produces five non-varying cluster centroids, often referred to as “dynamic states” or dFNC states (Figure 2) reported in previous studies (Damaraju et al., 2014; Rashid et al., 2014, 2019; Yu et al., 2015; Miller et al., 2016b,c; Yaesoubi et al., 2017; Espinoza et al., 2019b; Mennigen et al., 2019; Weber et al., 2020; Faghiri et al., 2021; Fu et al., 2021). We mention them because they are referenced later in the results section of this paper. To distinguish time-blind non-varying states from EVOdFNCs, we will refer to them as non-varying “snapshot” dFNC states (SNAPdFNC). There are two modularized SNAPdFNC states dominated by strong positive auditory-visual-sensorimotor (AVSM) connectivity: one (State 1) has strong negative connectivity between the default mode network (DMN) and the AVSM networks; the other (State 2) presents weak DMN-to-AVSM connectivity. HCs spend more time in both of these AVSM-dominant modularized states than the patients do. A third modularized state (State 3) is defined by a contrast between strong negative connectivity between the DMN and the rest of the brain, with diffuse positive connectivity in all other blocks of the connectome. We will use the shorthand, *DMNneg*, in future references to this particular modularized SNAPdFNC state, which is more occupied by patients than controls. The other two SNAPdFNC states (States 4 and 5) show much less modularity: one (State 4) is diffusely hyperconnected and more occupied by controls; the other (State 5) is diffusely disconnected and more occupied by patients (Damaraju et al., 2014).

### Planar Embedding

We apply a MATLAB implementation (McInnes et al., 2018; Meehan et al., 2020) of UMAP to embed all 1, 081-dimensional dFNCs into the plane. Although both UMAP and t-distributed stochastic neighbor embedding (t-SNE) preserve high-dimensional local structure in the lower-dimensional embedding, UMAP holds onto more global structure than t-SNE, exhibits greater stability across runs, and is also significantly more efficient (see Supplementary Files for experiments clarifying the differences between UMAP and *t*-SNE and the role of parameters in stabilizing the embedding). Key user–chosen parameters in UMAP are the *number of nearest-neighbors* (n_neighbors) over which the high-dimensional uniformized neighborhoods are defined, and the *minimum distance* (min_dist) which parameterizes proximity in the low-dimensional embedding. The stability and “reasonableness” of UMAP embeddings, especially with respect to preservation of global structure, can be sensitive to initialization (Kobak and Linderman, 2021), although our specific data were relatively stable under re-initialization of the algorithm and we further stabilized the final embedding by averaging over multiple runs (Supplementary Figure 4). Because dFNCs are computed on sliding windows that advance one TR at a time, they exhibit considerable temporal smoothness in their native high-dimensional space (mean elementwise squared distance between successive dFNC measures is less than 0.0006). The high-dimensional trajectories are also somewhat “densely packed,” i.e., for any fixed dFNC observation, the average mean squared elementwise distance between that observation and its 10,000 nearest neighbors *from different subjects* is less than 0.01. The ideal embedding for our purposes would preserve intra-subject trajectory continuity and inter-subject trajectory proximity. This within-subject smoothness is an intrinsic feature of the actual dynamics, which we experimentally optimized UMAP’s two main parameters (n_neighbors = *25*; mindist = 0.75) toward conserving in the planar embedding (Supplementary Figure 3). It is worth quickly noting that linear dimension-reduction methods such as PCA or ICA produced diffuse two-dimensional (2D) clouds lacking intra-subject temporal continuity (Figure 3), whereas t-SNE performed more than 200-fold more slowly than UMAP on these data and, thus, was not practical for achieving a planar embedding of the entire dataset.

Because the UMAP embeddings are not fully deterministic, we run the algorithm *R = 25* times on the input dFNC data *𝒟* and use the average $\hat{ℰ}\left(𝒟\right)=\left\{\left(\frac{1}{R}\sum_{r=1}^{R}x_{r}\left(t\right),\frac{1}{R}\sum_{r=1}^{R}y_{r}\left(t\right)\right),s=1,2,..,n,t=1,2,\ldots ,T\right\}$, of the *R* individual 2D embeddings ${ℰ}_{r}\left(𝒟\right)=\left\{\left(x_{r}^{s}\left(t\right),y_{r}^{s}\left(t\right)\right),s=1,2,\ldots ,n,t=1,2,\ldots ,T\right\}$. This averaging procedure stabilizes the embedding but also de-densifies the group level geometry (Supplementary Figure 4), reducing its qualitative fidelity to the native high-dimensional setting.

### Linearized 2D Dynamic Functional Network Connectivity Trajectory Segments and Gradient Exemplars

**FIGURE 10:**
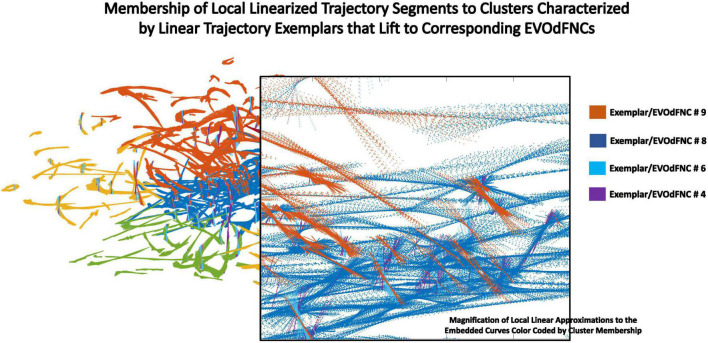
Zooming in on a box within the display (transparent background) of local linearizations of embedded curves (LTSs) colored according to the 2D linear trajectory exemplar cluster to which they belong. Within the same region, the individual LTSs can belong to different exemplar clusters based on slope.

Because of our choice of UMAP parameters within intervals that preserve intra-subject trajectory continuity, the majority of each subject’s high-dimensional dFNC trajectory Γ(*t*) = (*v*_1_(*t*), *v*_2_(*t*), … , *v*_1081_ (*t*)) embeds, under the *r^th^* run of UMAP, into an approximately continuous segment γ_r_(*t*) = (*x*_*r*_ (*t*), *y*_*r*_ (*t*)) in 2D (Figure 4). Continuity is preserved under summation, so the average $\hat{\gamma }\left(t\right)=\left(\hat{x}\left(t\right),\hat{y}\left(t\right)\right)=\left(\frac{1}{R}\sum_{r=1}^{R}x_{r}\left(t\right),\frac{1}{R}\sum_{r=1}^{R}y_{r}\left(t\right)\right)\in \hat{ℰ}\left(𝒟\right)$ will also be continuous. We collect all continuous trajectory sub-segments (CTSs) ${\hat{\alpha }}_{j}\left(\hat{\gamma }\right)=\left\{\hat{\gamma }\left(t\right),t\in \left[t_{j},t_{j}+\tau \right]\right\},j=1,2,\ldots ,T-\tau$ along $\hat{\gamma }\left(t\right)$ of temporal duration τ = 44 (twice the window length used to estimate the dFNC observations in *𝒟*). Each ${\hat{\alpha }}_{j}\left(\hat{\gamma }\right)$ can be approximated by a line *L_j_*, yielding a reduced characterization $\left({\hat{x}}_{j},{\hat{y}}_{j},{\hat{m}}_{j},{\hat{\ell }}_{j}\right)$ of $\hat{\alpha_{j}}\left(\hat{\gamma }\right)$ in terms of its length ${\hat{\ell }}_{j}=\max_{t,t^{\prime }\in \left[t_{j},t_{j}+\tau \right]}{||\hat{\gamma }\left(t\right)-\hat{\gamma }\left(t^{\prime }\right)||}_{2}$ , its geometric midpoint $\left({\hat{x}}_{j},{\hat{y}}_{j}\right)$, and the slope ${\hat{m}}_{j}$ of its linearization *L_j_* (Figure 5). The linearized trajectory segment (LTS) triples: $\left({\hat{x}}_{j},{\hat{y}}_{j},{\hat{m}}_{j}\right)$ are then clustered (Figure 10) with k-means [MATLAB implementation, squared Euclidean distance, 2,000 iterations, 250 repetitions, slope bounded between positive and negative max ({|*x*|, |*y*|}) to keep arbitrarily large near-vertical slopes from dominating the spatial (x, y) components of the clustering] where the number of clusters, *K = 10*, was chosen according to the elbow criterion. From each of the *K = 10* LTS cluster centroids $\left({\hat{x}}_{j},{\hat{y}}_{j},{\hat{m}}_{j}\right)$, we induce a 2D line segment of length *d_i_* (equal to the mean length of all LTSs in that cluster) and slope ${\hat{m}}_{i}$ centered at $\left({\hat{x}}_{i},{\hat{y}}_{i}\right)$ (Figures 4, 5). These segments are, roughly speaking, *gradients* of the CTSs, which are, in turn, averages of continuous embedded segments of the high-dimensional dFNC dynamics. The collection of segments induced by LTS cluster centroids will be called *linear trajectory exemplars* or just *exemplars*. Although UMAP is a non-linear dimension reduction algorithm, implying, among other things, that line segments in the planar embedding are not necessarily linear segments in the source domain – the line segments moving along dominant embedded curve directions, capturing local spatial trends from *t* to *t + 1*, will have non-linear inverse images that similarly reflect local trends in the high-dimensional dynamics as locality is highly preserved under UMAP.

### Prototype High-Dimensional Evolving Dynamic Functional Network Connectivity Basis States (EVOdFNCs)

**FIGURE 11:**
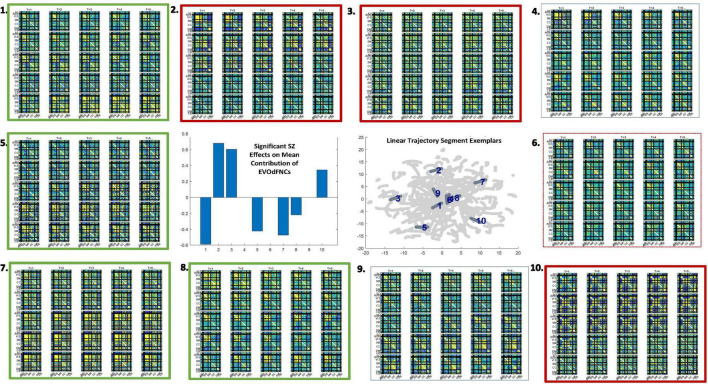
There are pervasive group differences between schizophrenia patients and controls in representational importance of the *K* = 10 EVOdFNCs; thick red (respectively, thick green) boxes designate significant positive (respectively, negative) association with SZ after correction for multiple comparisons; thin dashed red box designates significant (*p* < 0.025) positive association with SZ that is not significant after correction for multiple comparisons. Omitted colorbar is bounded by [−0.3, 0.3].

Because UMAP is not straightforwardly invertible, the 2D linear trajectory exemplars above cannot easily be mapped back directly into the native dimension of the dFNC space. To obtain the high-dimensional dFNC trajectory segment ϒ of integer temporal duration τ corresponding to a given 2D linear trajectory exemplar ϒ, (i.e., the “data driven inverse” of υ), we average the high-dimensional observations corresponding to the *n = 25* nearest 2D neighbors of each of τ evenly spaced points along υ (Figure 7). The points utilized are the 25 *spatial* nearest embedded neighbors to each of the τ evenly spaced points along *v*. For the purpose of inverting planar points not associated with samples, the slope of the linear trajectory segments centered at these points are not taken into account because the embedding itself is working in spatial terms exclusively and its “data-driven” inverse should work in the same terms as the embedding. The number of 2D neighbors used for this inversion of the UMAP embedding, *n = 25*, was chosen to match the number of nearest neighbors parameter that we employed for running the UMAP algorithm. The operation thus described effectively “lifts” a localized 2D linear trajectory exemplar back into high-dimensional dFNC space. The 2D linear trajectory exemplars are, by construction, concentrated in more densely occupied parts of the plane, and the continuity preserving parameterization of UMAP encourages the high-dimensional data-driven inverse of each 2D linear trajectory exemplar to exhibit naturalistic smoothness (Figure 11). These high-dimensional inverse images of the 2D linear trajectory exemplars are each multiframe representations of evolving functional network connectivity (EVOdFNCs).

### Expressing Observed High-Dimensional Dynamic Functional Network Connectivity Trajectories in Terms of EVOdFNC Basis States

The high-dimensional EVOdFNCs obtained from 2D exemplar linear trajectories are more properly viewed as representations of dominant directional trends than as a hard segmentation of the observed dynamics. We, therefore, employed them as basis objects through which to parameterize observed high-dimensional evolving connectivity dynamics. This is done by correlating successive time points (i.e., *t* = 1, 2, … , τ)of each length-τ window *w_i_* in a subject’s observed dFNC sequence with the corresponding set of *t^th^* time points from the *K* length-τ EVOdFNCs (Figure 8). For each *w_i_* and each *t* ∈ {1, 2, … , τ}, we identify the EVOdFNC *k* ∈ {1, 2, … , *K*} whose *t^th^* time point has the highest correlation with the *t^th^* time point of observed dFNC window *w_i_*, yielding a length-τ vector ρ = (ϱ_1_, ϱ_2_, … , ϱ_τ_), ϱ_*j*_ ∈ {1, 2, … , *K*}. The weight λ_*k*_(*w*_*i*_) ∈ [0, 1] on each *k* ∈ {1, 2, … , *K*} is the proportion of time points $\frac{\left\lfloor \left\{j\in \left\{1,2,\ldots ,\tau \right\}:\varrho_{j}=k\right\}\right\rfloor }{\tau }$ in *w_i_* that most resemble (are most correlated with) the same time point in EVOdFNC *k*. This approach both normalizes the weights in [0, 1] and accommodates the possibility that within any given time window of length τ, a subject’s dFNC sequence might contain subintervals that are best represented by the corresponding subinterval of different EVOdFNCs. From this, we obtain a *K*-variate time series of EVOdFNC weights $\Lambda ={\left\{\lambda_{k}\left(w_{i}\right)\right\}}_{k,i=1}^{K,\left(T-\tau \right)}$ for each subject (Figure 8, bottom left), capturing the relative RI of each EVOdFNCs to successive length-τ intervals of the subject’s observed high-dimensional dFNC trajectory. To be clear, the weight attached to a given EVOdFNC *k*, for a given length-τ window *w* of observed dFNCs, will be λ ∈ [0, 1], denoting the proportion of the τ dFNC observations in *w* that are more correlated to the corresponding frame in EVOdFNC *k* than they are to the corresponding frames in other EVOdFNCs. This assigns weight to each EVOdFNC according to the level at which its ordered sequences of frames represent (correlationally) represent ordered subsequences within the window of dFNC observations.

### Obtaining *Meta*-EVOdFNCs From Multivariate EVOdFNC Time Series

Because EVOdFNCs are inverse images of 2D segments in a planar embedding of very high-dimensional data, using weighted combinations of the EVOdFNCs (as opposed, e.g., to a binary “occupancy” approach) is arguably a better strategy for capturing the evolving variability of high-dimensional dFNC observations. Toward this end, we cluster (across all windows and subjects) the time-indexed weight vectors from the multivariate EVOdFNC time series into *M = 10* clusters (elbow criterion, MATLAB’s k-means implementation, squared Euclidean metric, 2,000 iterations, and 500 repetitions). We then induce *M meta*-EVOdFNCs as centroid-weighted sums of the basis EVOdFNCs (Figure 9). Subject-specific *occupancy rates* of each *meta-*EVOdFNC are computed as the fraction of the subject’s time-indexed weight vectors that belong to the cluster whose centroid defines the *meta*-EVOdFNC.

### Statistical Modeling

All reported SZ effects are from a multiple regression on gender, age, head motion (mean frame displacement), and SZ. Reported positive PANSS symptom score effects are from a multiple regression on the six positive PANSS symptoms (delusions, grandiosity, hallucinations, suspiciousness/persecution, preoccupation, and unusual thought) from the Lindenmayer five-factor PANSS model (Lindenmayer et al., 1995), in addition to age, gender, and head motion. The purpose of this model is to identify the effect of each positive symptom corrected for the contributions of the others, in a manner indifferent to, i.e., summing over, the various profiles of negative and general symptoms that subjects might manifest. Whereas negative symptoms in the SZ subjects analyzed here are highly intercorrelated [mean correlation between negative symptom pairs = 0.31 (std. dev = 0.12)], positive symptoms are not [mean correlation between positive symptom pairs = 0.12 (std. dev = 0.13)]. Schizophrenia effects are only reported when significant at the *p* < 0.05 level *after* correction for multiple comparisons. All displayed PANSS symptom effects are significant at the *p* < 0.05 level and remain significant at this level after correction for multiple comparisons where specifically indicted.

## Results

### Representational Importance of EVOdFNCs: Effects of Schizophrenia and Positive Symptoms

We found widespread schizophrenia effects on the RI of each of the 10 EVOdFNCs in subject data (Figure 11). The evolving motifs fluidly move through transient states of connectivity that resemble familiar formations obtained from the basic time-blind clustering into single transiently realized connectivity patterns (Figure 2). Consistent with published results (Damaraju et al., 2014; Miller et al., 2016b; Yaesoubi et al., 2017; Espinoza et al., 2019b; Rashid et al., 2019) on occupancy rates of time-blind SNAPdFNC states, we found the following: strongly modularized and hyperconnected patterns feature more prominently in EVOdFNCs with greater RI in controls (1, 5, 7, and 8) and in EVOdFNCs whose RI in controls is not statistically distinguishable from that in patients (4, 6, and 9); weak connectivity and modularized negative DMN-to-other (DMNneg) patterns (2 and 3) feature more prominently in EVOdFNCs with significantly higher RI in patients. A novel modularized pattern of functional organization, not seen in time-blind SNAPdFNC states, appears in EVOdFNC 10, which features a persistent stretch of strong modularized negative SM-VIS/CC/DMN connectivity. EVOdFNC 10 has significantly greater RI in patients, and its distinctive modularity eventually dissolves into unstructured weak connectivity. EVOdFNC 7, with greater RI in controls, transitions from a weakly connected negative DMN-to-other structure (more characteristic of patients in time-blind analysis, e.g., SNAPdFNC State 3) into another novel pattern that is strongly modularized with positive DMN-VIS/SM connectivity. EVOdFNC 2 (+SZ, RI) and EVOdFNC 5 (+HC, RI) show roles for disconnected periods leading into and out of modularized structures that are more characteristic of SZ and HC, respectively. EVOdFNC 2 (+SZ, RI) also exhibits a transition from something more like SNAPdFNC State 1 (+HC, OCR) to SNAPdFNC State 3 (+SZ, OCR), presenting a path from strong AVSM-focused modularity (+HC, SNAPdFNC OCR) to the DMNneg configuration (+SZ, SNAPdFNC OCR) to weak connectivity (+SZ, SNAPdFNC OCR).

**FIGURE 12:**
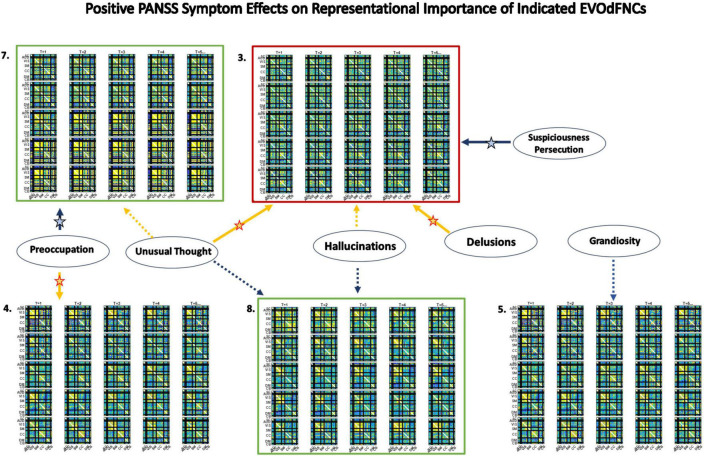
Significant positive symptom effects on the representational importance of EVOdFNCs in high-dimensional dFNC dynamics. Thick arrows with stars indicate effects that are significant after correction for multiple comparisons; thin dashed arrows indicate effects that are significant with *p* < 0.05, but not after correction for multiple comparisons. Yellow lines denote positive effects. Blue lines denote negative effects. EVOdFNC 3, the low-contrast, low-dynamism DMNneg patterned state is a locus of positive symptom effects: RI elevated by Hallucinations, Delusions and Unusual thought, and suppressed by Suspiciousness/Persecution. EVOdFNC 7, dynamically changing from a low-contrast DMNneg pattern to an AVSM-dominant hyperconnected pattern is more important in HCs than SZs, but among SZs with high levels of Preoccupation its importance is further significantly suppressed and elevated in those with high levels of Unusual Thought. Note that positive symptoms exhibit no significant (*p* < 0.05, uncorrected) effects on the occupancy rates of the SNAPdFNCs.

EVOdFNCs 1 and 8 (both +HC, RI) show the role of disconnected periods in transitioning between AVSM-centered modularity and hyperconnectivity in healthy people: in EVOdFNC 1, we see modularity dissolving into diffuse dysconnectivity, which then uniformly inflates toward diffuse hyperconnectivity, whereas, in EVOdFNC 8, diffuse hyperconnectivity gradually erodes toward greater dysconnectivity away from the AVSM core and then starts manifesting negative DMN/CC-AVSM connectivity from a substrate of dysconnectivity. Although EVOdFNCs 2 and 3 both have greater RI in patients and both contain patterns similar to SNAPdFNC State 3 (+SZ, OCR), it is the weaker, less dynamically varying EVOdFNC 3 whose RI is significantly further elevated in more delusional patients (Figure 12). The RI of EVOdFNCs 4 and 9 is not significantly affected by subject diagnosis, but they reveal patterns of functional organization that do not appear in time-blind dFNC clustering. Specifically, EVOdFNC 4 evolves from strong AVSM-centered modularity (+HC, SNAPdFNC OCR) through a DMNneg configuration (+SZ, SNAPdFNC OCR) connectivity into a modularized pattern, in which intra-AVSM and intra-CC connectivity is at approximate parity. EVOdFNC 9 passes through a stage in which intra-CC connectivity is even stronger than intra-AVSM connectivity. Transient periods of whole-brain connectivity where connectivity strength within the CC domain equals or exceeds that within AVSM domain block do not appear in time-blind dFNC clustering of these data. EVOdFNCs 4 and 9 both have strong population-wide RI (Supplementary Figure 2) indicating that these patterns play a meaningful role in dynamic connectivity despite not appearing in SNAPdFNC clusters centroids.

**FIGURE 13:**
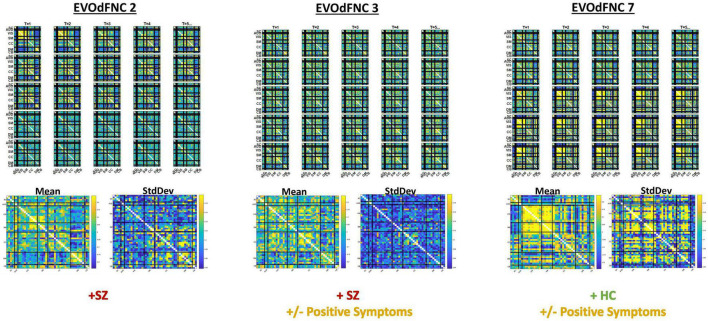
Three EVOdFNCs (2, 3, and 7) start with intervals characterized by the modularized DMNneg patterning defined by SNAPdFNC State 3, a state that is more occupied by SZ patients. All three EVOdFNCs start with a DMNneg type of pattern, but each presents a different dynamic context. EVOdFNC 2 (top left) exhibits a high-contrast DMNneg pattern that dissolves into diffuse dysconnectivity. EVOdFNC 3 (top middle) presents a persistent, unvarying low-contrast DMNneg pattern. EVOdFNCs 2 and 3 have very similar temporal means (displayed below each EVOdFNC), with distinguishing characteristics requiring representation of their temporal evolution. Only EVOdFNC 3 has significant positive symptom effects. In EVOdFNC 7, the DMNneg pattern is part of a dynamic progression leading to AVSM-dominant hyperconnectivity. Although DMNneg patterning is significantly associated with SZ in time-blind SNAPdFNC analysis, EVOdFNC 7 not only has higher RI in HCs but also is sensitive to positive symptom levels in SZ patients.

Three of the 10 EVOdFNCs start with intervals characterized by modularized DMNneg patterning, i.e., strong negative connectivity between DMN and all other brain areas with positive connectivity everywhere else. The SNAPdFNC state with this pattern is more occupied by SZs than HCs. Two of the EVOdFNCs (2 and 3) have higher RI in SZs, one (EVOdFNC 7) in HCs. Two have significant positive PANSS symptom effects (Figure 12): One of these (EVOdFNC 3) has higher RI in SZs, and the other (EVOdFNC 7) has higher RI in HCs. Although all three EVOdFNCs pass through a DMNneg type of pattern, each embeds this pattern in a different dynamic context (Figure 13). EVOdFNC 2 presents a high-contrast DMNneg pattern dynamically dissolving into diffuse dysconnectivity. Its temporal mean (bottom left) resembles that of EVOdFNC 3, which features persistent, low-contrast, unvarying DMNneg. Both EVOdFNCs 2 and 3 have higher RI in SZs, but only the lower contrast, less dynamic version, EVOdFNC 3, has significant further relationships to positive symptoms within the patient population (Figure 12). The temporal averages of EVOdFNCs 2 and 3 (Figure 13, bottom row) are very similar, suggesting that the differential sensitivity of EVOdFNCs 2 and 3 to positive symptoms resides in their temporal patterning. Finally, although, in time-blind SNAPdFNC analysis, the DMNneg pattern is significantly more occupied by SZs, we found EVOdFNC 7 (Figure 13, top right) featuring DMNneg, leading into AVSM-dominant, lightly modularized hyperconnectivity to have significantly higher RI in HCs and also to be sensitive to positive symptoms in SZs (Figure 12).

### Occupancy Rates of *Meta*-EVOdFNCs: Effects of Schizophrenia and Positive Symptoms and Relationship to EVOdFNCs

**FIGURE 14:**
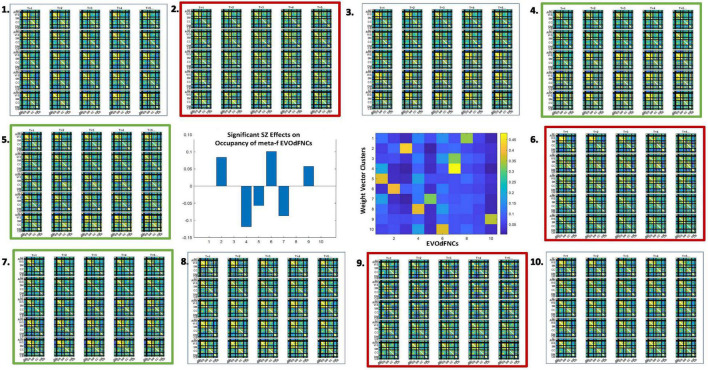
As with representational importance of EVOdFNCs, we see many significant group differences between schizophrenia patients and controls in the occupancy rates of meta-EVOdFNCs; thick red (respectively, thick green) boxes designate significant positive (respectively, negative) effect of SZ on meta-EVOdFNC occupancy rate; all displayed effects are significant at the *p* < 0.05 level after correction for multiple comparisons. The cluster centroids of the EVOdFNC weight vectors from which the meta-EVOdFNCs (middle row, column 3) are induced tend to focus the majority of weight on one EVOdFNC, so there is evident resemblance between meta-EVOdFNCs and the EVOdFNCs. Note that positive symptoms exhibit no significant (*p* < 0.05, uncorrected) effects on the occupancy rates of the SNAPdFNCs.

**FIGURE 15:**
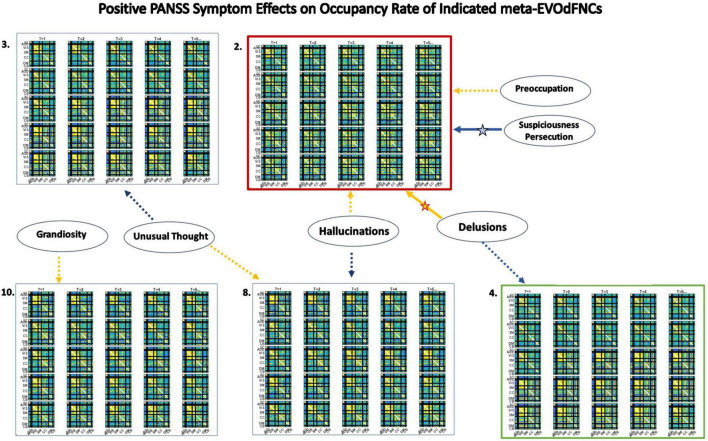
Significant positive symptom effects on the occupancy of meta-EVOdFNCs, each a weighted combination of EVOdFNCs. Thick arrows with stars indicate effects that are significant after correction for multiple comparisons; thin dashed arrows indicate effects that are significant with *p* < 0.05, but not after correction for multiple comparisons. Yellow lines denote positive effects. Blue lines denote negative effects. Consistent with results on representational importance of EVOdFNCs, meta-EVOdFNC 2 which is heavily weighted on EVOdFNC 3 is a locus of positive symptom effects, including positive effects of delusions and negative effects of suspiciousness/persecution that remain significant after correction for multiple comparisons. Note that positive symptoms exhibit no significant (*p* < 0.05, uncorrected) effects on the occupancy rates of the SNAPdFNCs.

The 2D linear exemplars that lift to high-dimensional EVOdFNCs are relatively sparse in the planar embedding. Many local linearizations of embedded subject dFNC trajectories are not geometrically proximal to an exemplar or are not directionally aligned with the nearest exemplar. Moreover, the local linearizations are only approximations to the embedded curves, which, in turn, are imperfect representations of the high-dimensional ground truth. This suggests that a multivariate characterization of the dynamics in terms of replicable patterns, i.e., clusters, of concurrent multivariate EVOdFNC RI would induce meta-EVOdFNCs (Figure 9) that capture more of the high-dimensional data variability than the RI of individual EVOdFNCs. In practice, for the data evaluated in this study, the resulting weight-vector centroids (Figure 14, center right) were each highly concentrated on one EVOdFNC, yielding meta-EVOdFNCs that strongly resemble the dominantly weighted EVOdFNC in the corresponding weight-vector cluster centroid (Figures 11, 14). However, although focused on one dominant EVOdFNC, the meta-dFNCs include content from all EVOdFNCs, which allows for more flexible representation of the data when warranted. Moreover, the occupancy rate forces a harder segmentation than RI, yielding slightly different results. Delusions, for example, show positive effects on the RI of EVOdFNC 3 and on the occupancy rate of meta-EVOdFNC 2 (Figure 15), which is heavily weighted toward EVOdFNC 3. Delusions, however, also exhibit negative effects on the occupancy rate of meta-EVOdFNC 4, which is heavily weighted on EVOdFNC 7 whose RI is not significantly affected by Delusions.

### Occupancy Rates of Underlying 2D Linear Exemplar Clusters: Effects of Positive Symptoms and Relationship to EVOdFNCs

**FIGURE 16:**
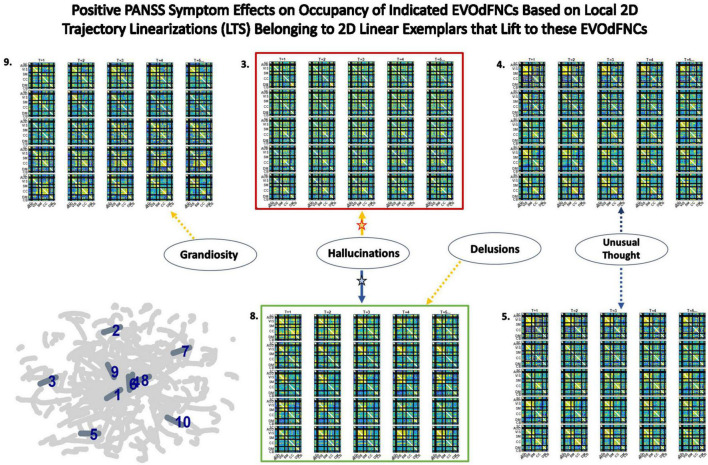
Five of the seven positive PANSS symptoms exhibited significant relationships with membership of local 2D trajectory linearizations to clusters defining the linear trajectory exemplars that lift to the displayed EVOdFNCs. Red (respectively, green boxes) around EVOdFNCs indicate positive (respectively, negative) SZ effects on their representational importance in observed high-dimensional dFNC trajectories. Yellow (respectively, blue) arrows point symptoms to EVOdFNCs with which they exhibit positive (respectively, negative) membership effects. Symptom effects are significant at the *p* < 0.025 level. The negative effect of Hallucinations with 2D exemplar #8, i.e., with EVOdFNC #8, remains significant at the *p* < 0.05 level after correction for multiple comparisons. Note that positive symptoms exhibit no significant (*p* < 0.05, uncorrected) effects on the occupancy rates of the SNAPdFNCs.

It is also possible to explore diagnosis and symptom effects directly in the 2D embedding, considering the linear exemplar cluster membership of each local linearization to an embedded subject trajectory (Figure 10). This approach “trusts” the embedding to distill some important relationships in the data while unavoidably distorting others. Surprisingly, there are no significant SZ effects on exemplar cluster membership of 2D local linearizations: nothing significant at the *p* < 0.05 level after correction for multiple comparisons, nor any raw uncorrected *p*-values less than 0.05. However, in the case of positive PANSS symptoms, we found a number of significant relationships between positive PANSS scores and linear exemplar cluster membership (Figure 16), one of which, the negative effect of Hallucinations on the linear exemplar that lifts to EVOdFNC 8, is significant at the *p* < 0.05 level after correction for multiple comparisons.

High-dimensional EVOdFNCs are inverses or lifts of 2D linear exemplars that summarize the planar embedding of group level high-dimensional dynamics. The exemplar membership of successive linear approximations to the embedded curves offers a “first pass” view of the alignment of subject connectivity dynamics with directional trends in the embedded data. As an embedding-intrinsic measure, exemplar cluster membership of 2D linear approximations is less concretely empirical than high-dimensional EVOdFNC RI. For positive SZ symptoms, this embedding-intrinsic point of view strengthens the uncorrected significance of, e.g., the Hallucinations effects on EVOdFNCs 3 and 8 while identifying several new relationships between positive symptoms and dimensionally reduced dFNC dynamics (Figure 16).

## Discussion

**FIGURE 17:**
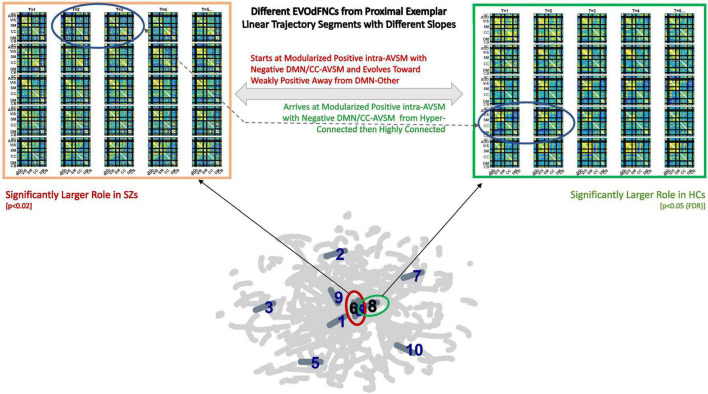
Top row shows two EVOdFNCs: leftmost in thick orange box is significantly (*p* < 0.025) more important in SZ before correction for multiple comparisons; the rightmost in the thick green box is significantly more important in HCs after correction for multiple comparisons. Note the similarity between the circled frames in both EVOdFNCs arising from the corresponding 2D linear trajectory exemplars that pass very near each other but have different slopes; the EVOdFNC with higher representational importance in healthy controls corresponds to the more horizontal exemplar (#8 in the thick green circle), whereas the nearby exemplar (#6 in the thick red circle) lifts to EVOdFNC 6, which has significantly higher RI in SZs. Although the EVOdFNCs in the top row are built from 2D linear trajectory exemplars that are proximal, especially near their endpoints, their differing slopes (i.e., angles of approach) cause them to represent dynamic trajectories sufficiently different as to be to represent high-dimensional dynamic contexts that are characteristic in one case of HCs and in the other of SZs.

Characterization and analysis of the time-varying resting state connectome continues to rely heavily on identifying a small number of fixed whole-brain connectivity patterns that manifest on timescales shorter than the full scan duration. The small set of fixed “states” is then then employed to model brain dynamics as a stationary Markov process, with the brain occupying and transitioning between this small set of fixed patterns. More sophisticated analyses of the complex dynamical processes that support human cognitive, emotional, executive, and motor functions require frameworks for characterizing and leveraging the fluidly varying high-dimensional dynamics presented by functional imaging modalities such as fMRI. Here, we introduce an approach that works from a data-driven inversion of the summary gradients in a planar embedding of the high-dimensional dynamics (Figure 17) to capture group-level multiframe evolving “movie-style” representations of dFNC (EVOdFNCs) in a large schizophrenia imaging study. We show that the method produces interpretable, naturalistic high-dimensional EVOdFNC states, whose contributions to HC and SZ dynamic connectivity differ significantly. The EVOdFNCs also expose distinct ways the two groups manifest and recede from certain characteristic organizational states of the connectome, e.g., the pattern in which DMN is anticorrelated with other networks and non-DMN networks are positively intercorrelated with each other (DMNneg).

Capturing characteristic longer sequences of evolving whole-brain connectivity exposes differences between patients and controls that reside in a complex joint feature space over temporal pacing, modular patterning, and overall strength of functional integration. Although the more traditional SNAPdFNC approaches do not elucidate variations in the dynamic connectome that correlate with positive symptoms of schizophrenia, this richer viewpoint shows, e.g., that a DMNneg pattern with weak contrast and limited temporal variation (EVOdFNC 3) is highly implicated in both SZ and its positive symptoms, whereas high-contrast DMNneg dissolving into dysconnectivity (EVOdFNC 2) strongly differentiates SZ from HC but displays no sensitivity to positive symptoms. Finally, EVOdFNC 7 that, like EVOdFNC 2, starts with high-contrast DMNneg but, instead of losing energy and modularity over time, gains connectivity strength while reconfiguring its modular patterning is negatively correlated with SZ while being both positively and negatively associated with positive SZ symptoms. Of the five EVOdFNCs that exhibit AVSM-dominant modularity, four (EVOdFNCs 1, 5, 7, and 8) are more representationally important in HCs and one (EVOdFNC 4) is highly important across the population (Supplementary Figure 2) but not significantly different between SZs and HCs. At the timescale being examined, τ = 44TRs (*88* s), the majority of the EVOdFNCs captured pass through several discernibly different configurations. This is true for all of the EVOdFNCs with significantly greater RI in HCs (1, 5, 7, and 8) and for two of the EVOdFNCs with significantly greater RI in SZs (2 and 10). Of the more static EVOdFNCs (3 and 6) (see Supplementary Figure 1), the one (EVOdFNC 3) that features consistent, low-contrast DMNneg plays a central role in positive symptom levels as well as SZ generally; the other (EVOdFNC 6) is consistently diffusely disconnected and has no significant role either in distinguishing patients from controls, or in connection with positive symptoms. Intervals of diffuse dysconnectivity are associated with significant group differences and symptom effects when they appear as subintervals in EVOdFNCs (1, 2, 5, and 10) that also have higher contrast intervals (see Supplementary Figure 1). The role of disconnected periods in the evolving connectomes of HCs and SZ differ in timescale and their role as “connective tissue” between higher-magnitude patterns of connectivity, suggesting that clinically relevant aspects of resting state connectivity dynamics are obscured by the field’s current overreliance on a simplifying Markov assumption. Fluidly evolving dynamic representations can also reveal important new connectivity patterns that are on the pathway from one familiar connectivity pattern from SNAPdFNC to another, e.g., the end of EVOdFNC 9 where intra-CC connectivity is stronger than AVSM connectivity and the negative connectivity between SM and part of the VIS with the rest of VIS in EVOdFNC 10.

**FIGURE 18:**
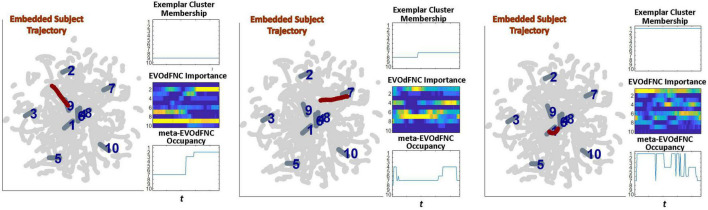
Three examples (leftmost panel in each subfigure) showing how proximity of subject embedded trajectories $\hat{\gamma }$ [maroon, superimposed with the 10 linear exemplars (dark gray, labeled in dark blue) over the full embedding (light gray)] are represented in terms of (right panel in each subfigure, top row) membership of local linear approximations to $\hat{\gamma }$ in clusters defining each of the 10 linear exemplars; (right panel in each subfigure, middle row) the representational importance of the high-dimensional EVOdFNCs corresponding to each 2D linear exemplar in the subject’s high-dimensional dFNC trajectory Γ, and (right panel in each subfigure, bottom row) the occupancy of meta-EVOdFNCs re-indexed to correspond with the EVOdFNC, which is most strongly weighted in each meta-EVOdFNC.

**FIGURE 19:**
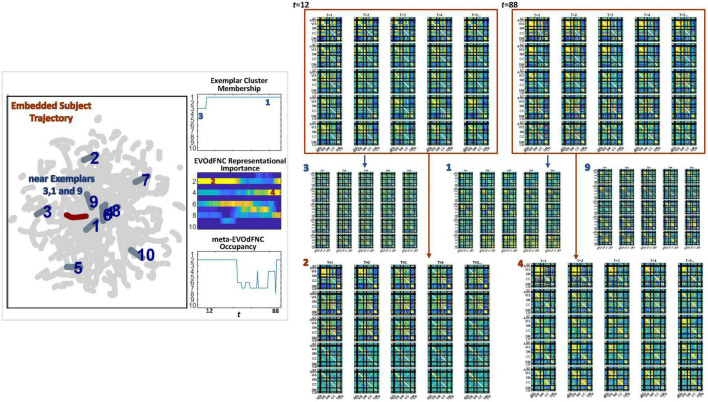
(Leftmost panel) One subject’s embedded trajectory $\hat{\gamma }$ (maroon) and the 10 linear exemplars (dark gray, labeled in dark blue) shown superimposed over the full embedding (light gray). The embedded trajectory passes near exemplars 3, 1, and 9. (Left panel, column 2, top) As expected, the local linearizations approximating $\hat{\gamma }$ belong to clusters defining exemplar 3 and then exemplar 1, both of which $\hat{\gamma }$ passes close to. (Left panel, column 2, middle) The time-varying representational importance of the high-dimensional EVOdFNCs corresponding to each linear exemplars in the subject’s high-dimensional dFNC trajectory Γ; the highest RI concentrates in EVOdFNCs 2 and 4, which are lifted from exemplars 2 and 4, neither of which is proximal to $\hat{\gamma }$; EVOdFNCs 3 and 1, corresponding to exemplars 3 and 1, have very low high-dimensional RI. (Right panel, top row) windows *w*_12_ and *w*_85_ starting at *t* = 12 and *t* = 85, which carry high RI from EVOdFNCs 2 and 4, respectively (Right panel, middle row), EVOdFNCs corresponding to exemplars 3, 1, and 9 that $\hat{\gamma }$ passes close to in the plane are not highly frame-wise correlated with Γ(*w*_12_) or Γ(*w*_85_), whereas (Right panel, bottom row) EVOdFNCs 2 and 4, which are most representationally important in Γ, do exhibit evidently strong framewise correlations with Γ(*w*_12_) and Γ(*w*_85_).

It is also important to mention that, while promising, this approach has a number of limitations that will benefit from further development. The method has a large number of parameters, from those that govern the UMAP embedding to the temporal scale (duration of EVOdFNCs) to the number of clusters at both the 2D exemplar and meta-EVOdFNC stages. There are different ways of assessing RI, each of which has benefits and drawbacks. Finally, there are multiple levels of results, emphasizing either the embedding itself, each high-dimensional EVOdFNCs individually, or weighted combinations of the EVOdFNCs. In this study, each meta-EVOdFNCs was strongly focused on one specific EVOdFNC, so results from the individual and multivariate levels tracked each other. The relationship between exemplar cluster membership, defined within the planar embedding, and the high-dimensional representational importance of EVOdFNCs to observed sequences of dFNCs is more complicated (Supplementary Figure 2), leading to some shared results at a statistical level (e.g., significant effects of hallucinations and delusions on exemplars/EVOdFNCs 3 and 8) with weaker relationships on a time point–by–time point basis (Figures 17–19). While acknowledging limitations, we believe this is an important first step toward more sophisticated analysis of high-dimensional functional imaging data, allowing researchers to more finely resolve the relationship of longer dynamic *processes* to human health and performance.

## Data Availability Statement

The data analyzed in this study is from phase III of the FBIRN study, which is a multi-phase, multi-institution schizophrenia study. Requests to access these datasets should be directed to Theodorus van Erp (tvanerp@uci.edu).

## Author Contributions

RM conceived of and designed the work reported here. RM, VV, and VC were involved with data analysis and interpretation. RM, VV, GP, and VC all participated in critical revisions and final approval of the submitted article.

## Conflict of Interest

The authors declare that the research was conducted in the absence of any commercial or financial relationships that could be construed as a potential conflict of interest.

## Publisher’s Note

All claims expressed in this article are solely those of the authors and do not necessarily represent those of their affiliated organizations, or those of the publisher, the editors and the reviewers. Any product that may be evaluated in this article, or claim that may be made by its manufacturer, is not guaranteed or endorsed by the publisher.
