# Supplementary material for: Multiframe Evolving Dynamic Functional Connectivity (EVOdFNC): A Method for Constructing and Investigating Functional Brain Motifs
Source: Front Neurosci. 2022 Apr 19;16:770468. doi: 10.3389/fnins.2022.770468 (PMC9063321; doi:10.3389/fnins.2022.770468)
Supplement: Supplementary file 1 [file Presentation_1.pdf]

Some figures addressing issues related to UMAP stability:

1. in the context of *densely packed continuous parametric curves* and

2. more typical high-dimensional greyscale image data

In all UMAPS shown in these slides, the *parameters are fixed at the values used in the paper*: **num_neighbors = 25**, **min_dist = 0.75**

Some figures addressing UMAP stability…in the context of ***densely packed continuous parametric curves***

<u>Lorenz attractor</u> as example of continuous, densely packed parametric curve in more than 2 dimensions

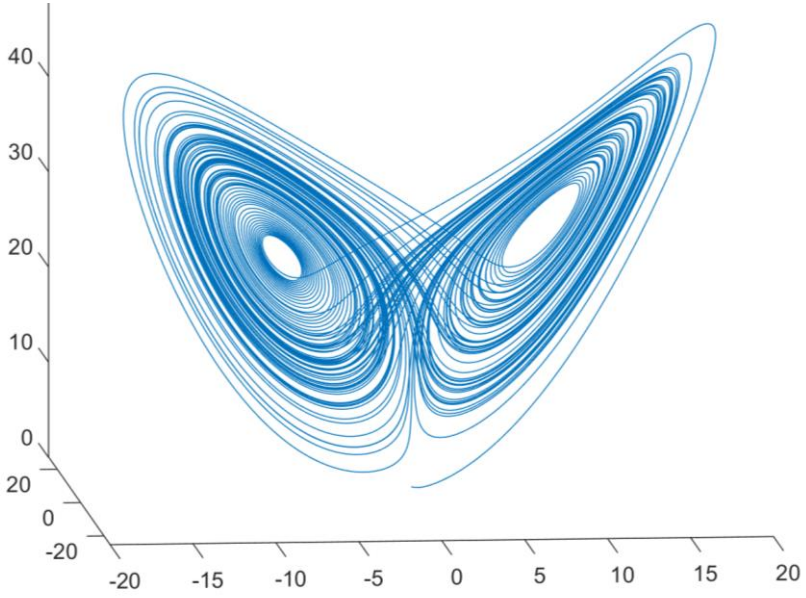

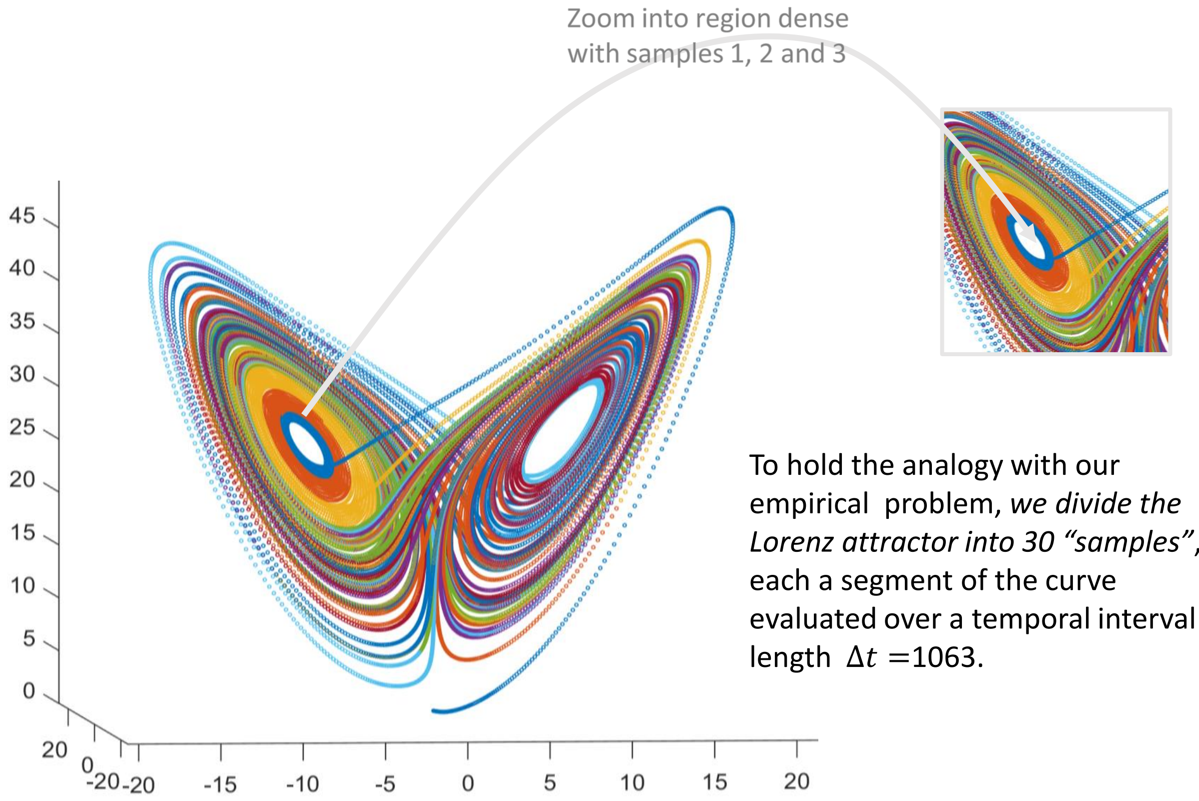

To hold the analogy with our empirical problem, *we divide the Lorenz attractor into 30 "samples"*, each a segment of the curve evaluated over a temporal interval of length Δt =1063.

20 planar embeddings of the Lorenz attractor using UMAP with fixed parameters (nNbrs=25, minDist=0.75). Furthermore, the order in which the attractor points are fed into UMAP is also held constant across the 20 runs, following the actual temporal ordering of the parametric curve.

Twenty UMAP embeddings of the Lorenz attractor under fixed parameters

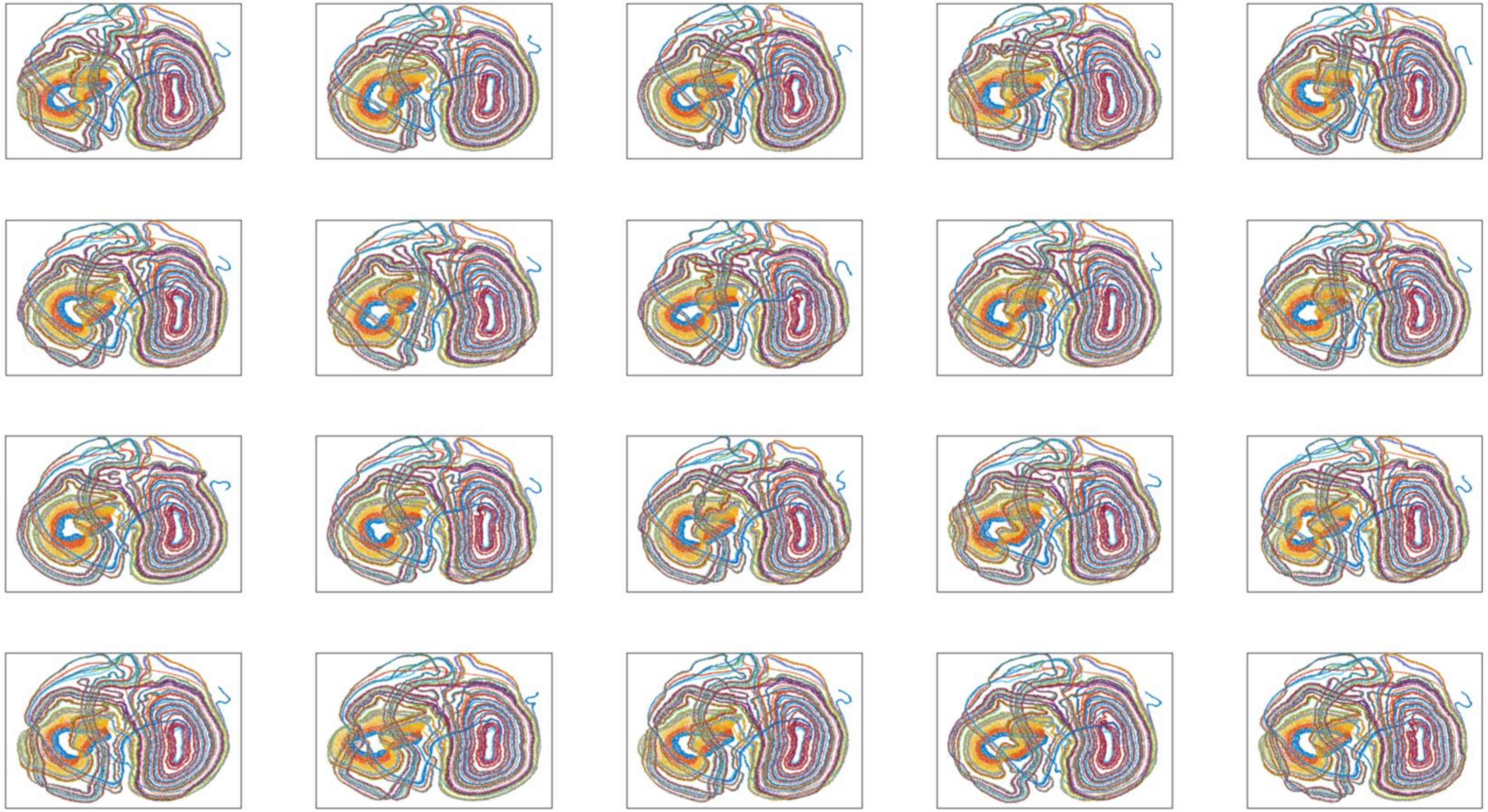

Averages of the embedded points over all runs

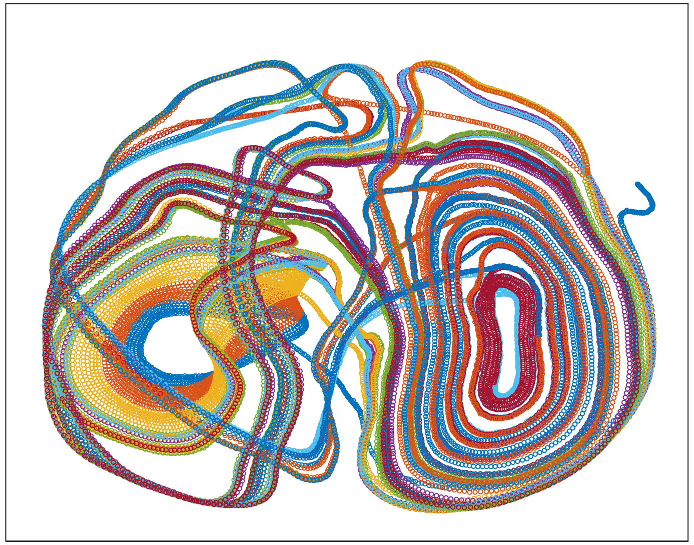

Note that the embedding preserves continuity, also density and directional congruence, e.g. the cluster of samples 1,2 and 3 in the bottom left

Also note that, while not identical, the runs have an aerial view type of stability that makes averaging points reasonable and doing so yields a sparsified, “cleaned up” version of the individual embeddings that roughly reflects direction and relative density of flow in each embedding, that in turn roughly reflects these features in the high dimensional dynamics

20 planar embeddings of the Lorenz attractor using UMAP with fixed parameters (nNbrs=25, minDist=0.75). In this case, the order in which the attractor points are fed into UMAP is permuted differently for each of the twenty runs

Twenty UMAP embeddings of the Lorenz attractor under fixed parameters: permuted samples

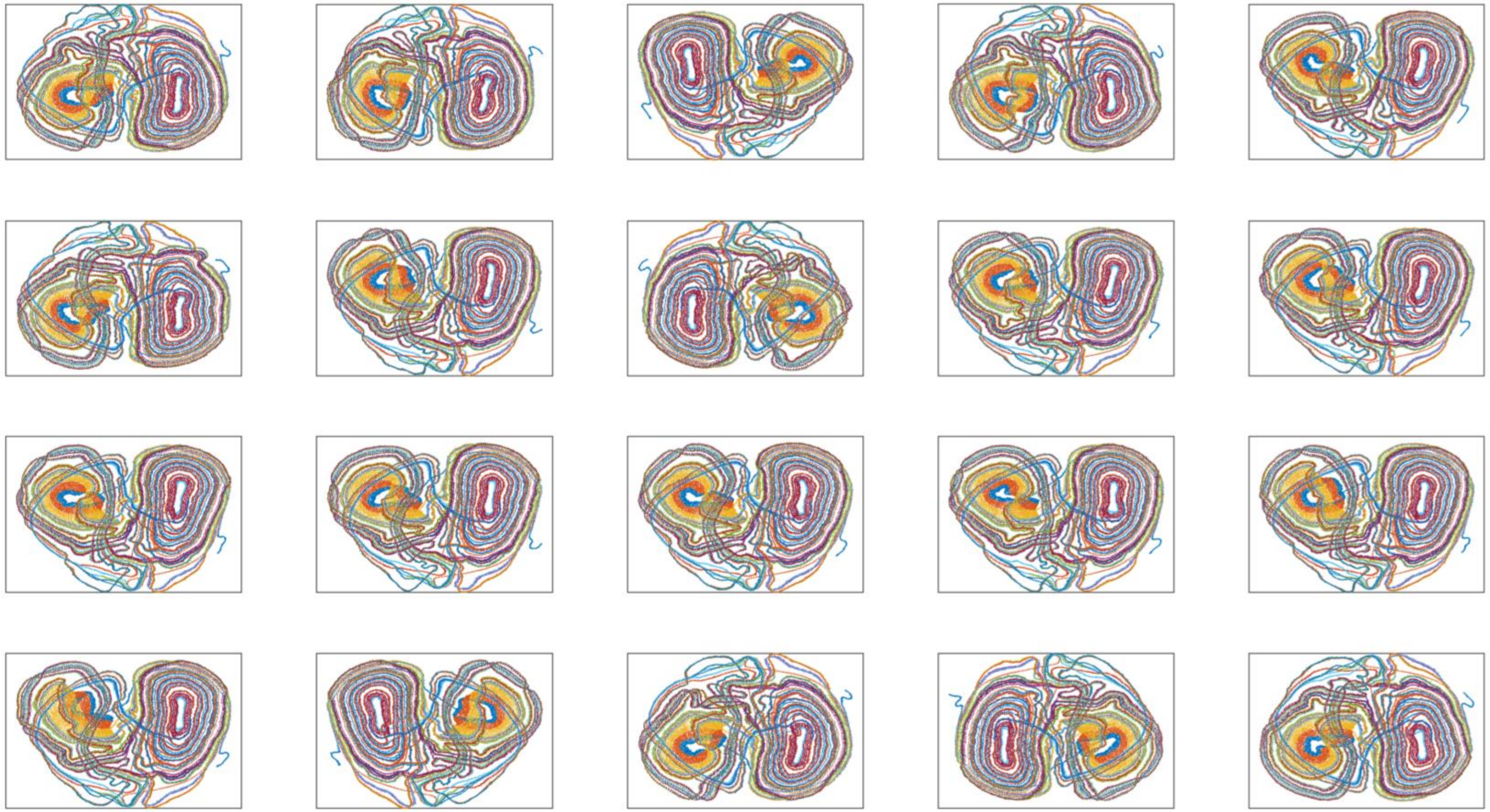

Averages of the embedded points over all runs

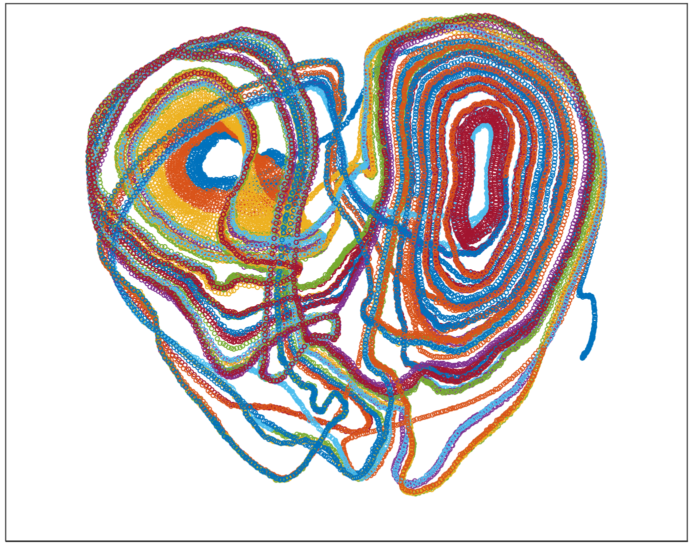

Note that the embedding preserves continuity, also density and directional congruence, e.g. the cluster of samples 1,2 and 3 (blue, red, gold)

Also note that, even when inputs are permuted, embeddings remain highly stable up to rotation. Most importantly, the inter-sample directional congruities, densities and proximities are preserved in these rotated embeddings

Final note: This specific set of permutations happens to produce 11 of 20 embeddings rotated so samples 1,2 & 3 are in the upper left, giving the cross-embedding pointwise average some structure here that would not hold in general.

Some figures addressing UMAP stability…in the less dynamically structured context of grayscale images

<u>Fashion MNIST</u> is a classification benchmarking dataset: 10 classes of clothing presented in 28 x 28 (784 vectorized dimensions) grayscale images

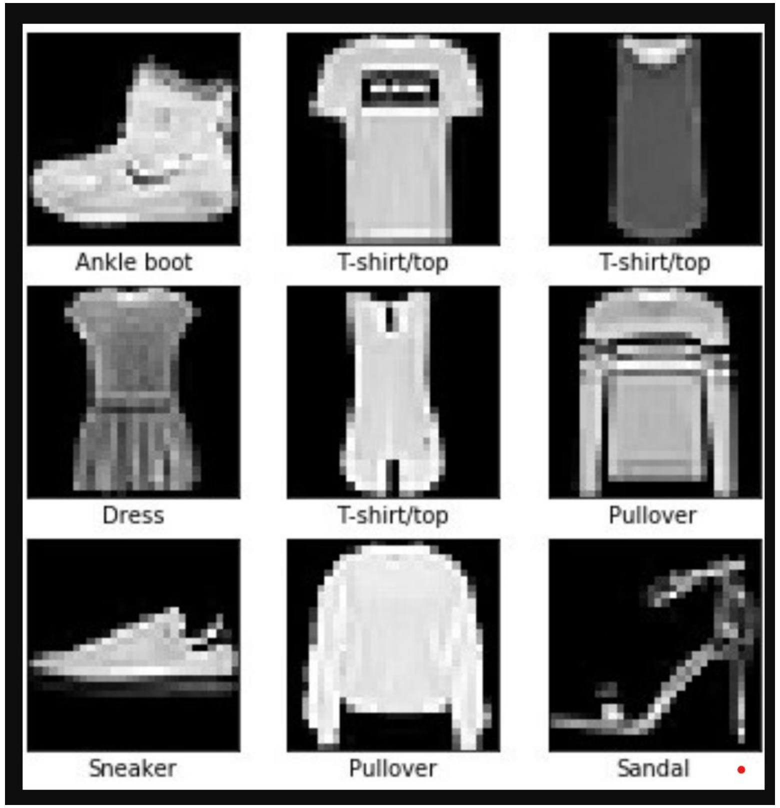

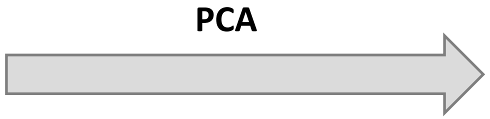

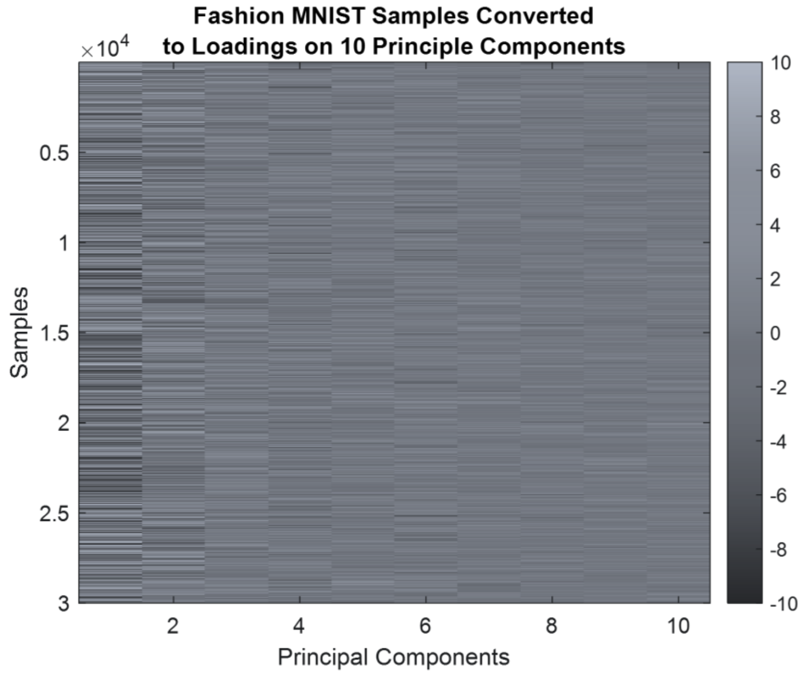

20 planar embeddings of Fashion MNIST using UMAP with fixed parameters (PCA reduced to 10D, nNbrs = 25, minDist = 0.75). The order in which the points are fed into UMAP is held constant across all 20 runs. No specific dynamics or continuity, but a general 10D data embedding problem.

Twenty UMAP embeddings of Fashion MNIST (10D PCA reduction) under fixed parameters

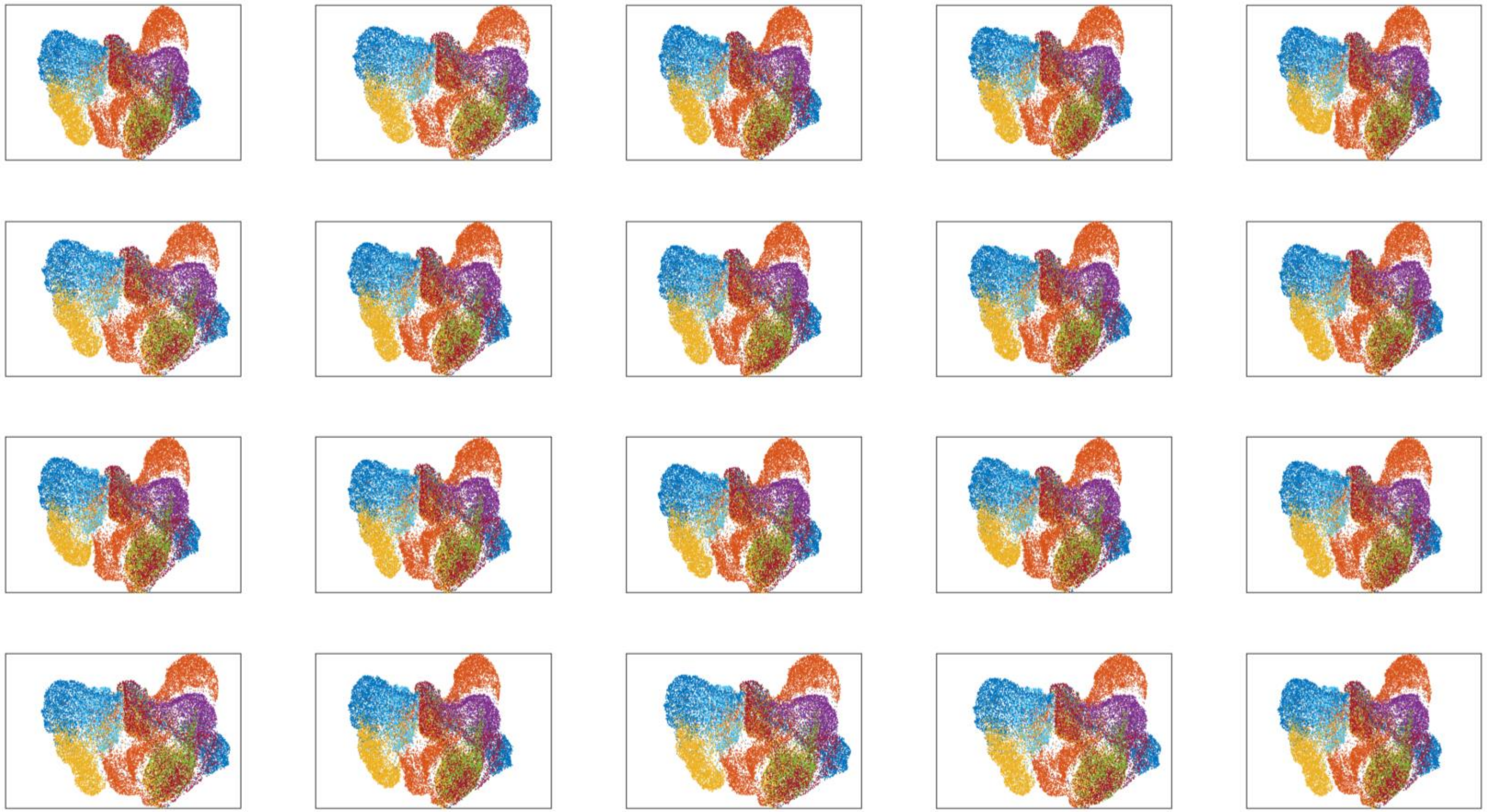

Averages of the embedded points over all runs

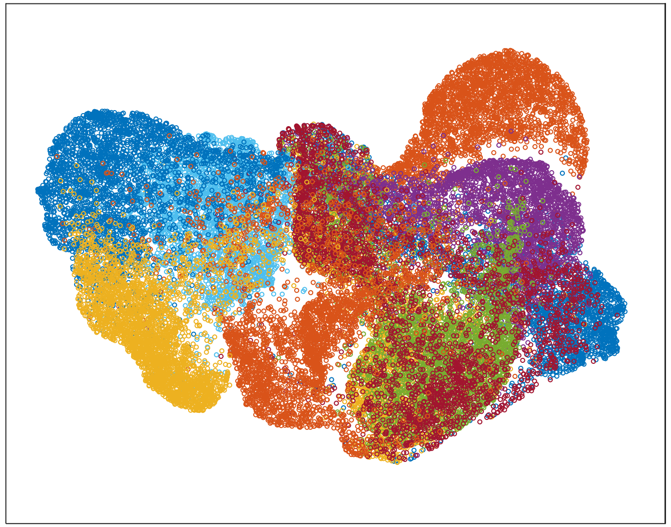

Again note that, while not identical, the runs exhibit a high level of stability that makes averaging points reasonable

20 planar embeddings of Fashion MNIST using UMAP with fixed parameters (PCA reduced to 10D, nNbrs = 25, minDist = 0.75). The order in which the points are fed into UMAP is permuted differently for each of the 20 runs.

Twenty UMAP embeddings of Fashion MNIST (10D PCA reduction) under fixed parameters

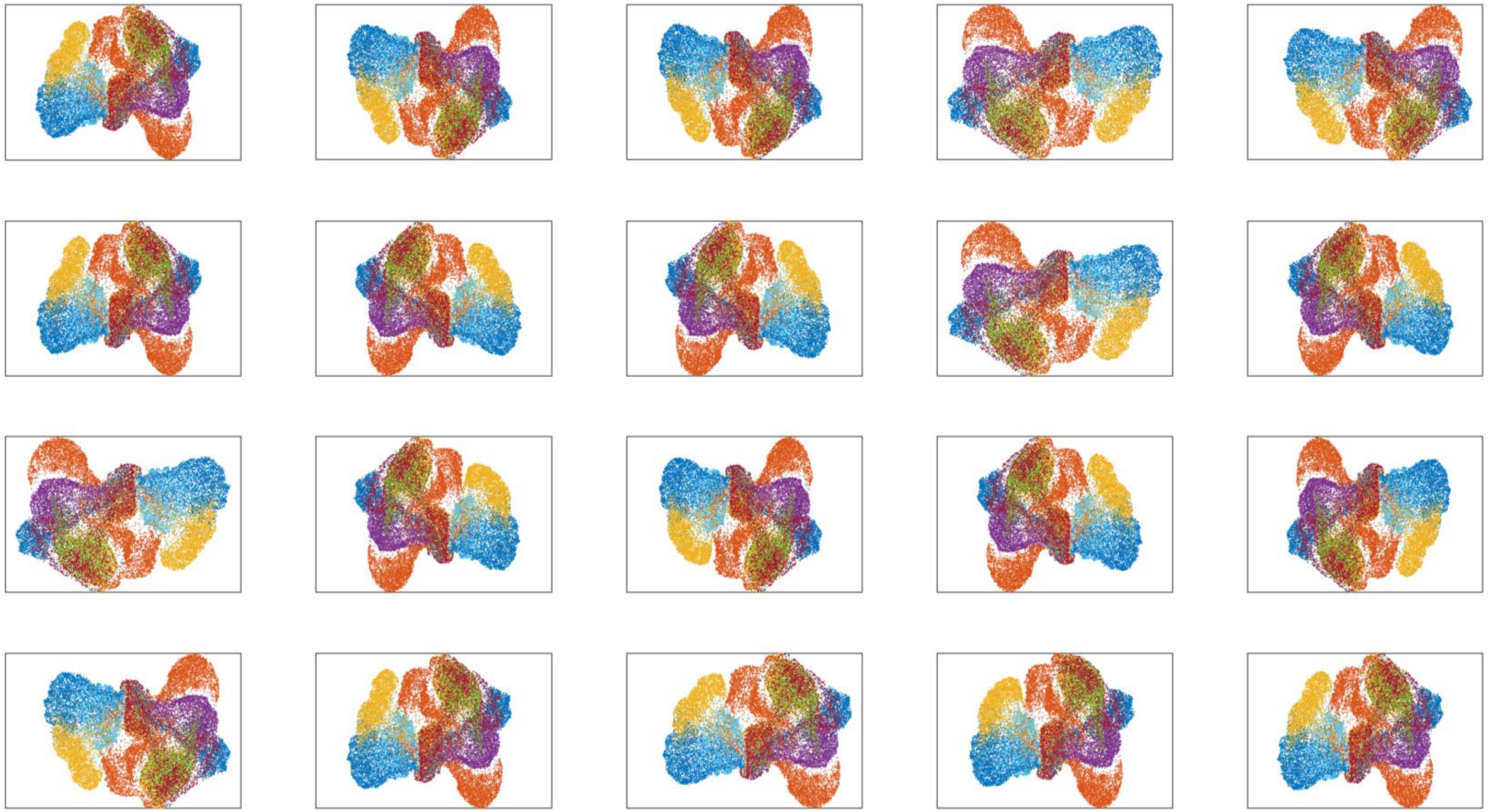

Averages of the embedded points over all runs

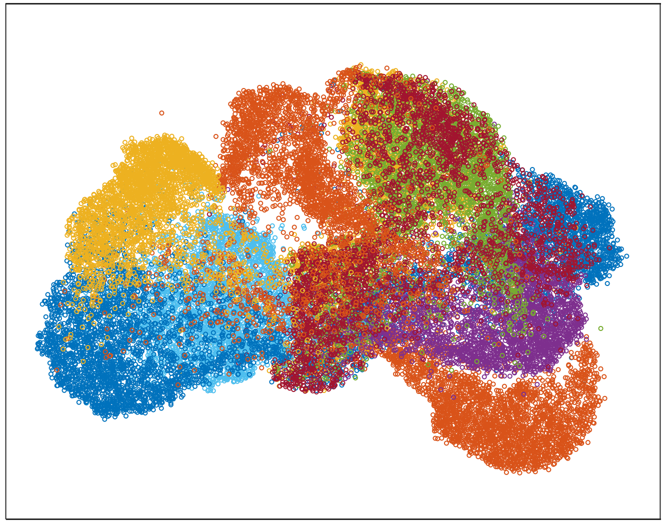

Note that, even when inputs are permuted, embeddings under different runs are highly stable up to rotation.

The rotation in which class 1 (t-shirts, red/orange) is on the bottom right appears in 6 or the 20 embeddings, slightly more than the other 3 rotations so the average maps more to that rotation. Axis ticks were omitted to clean up the display, but this omission does obscure the scaling effect of averaging: the x and y axes for individual embeddings are roughly [-12,12], while the averages are very compressed, with x and y axes spanning roughly [-1.25,1.25].

For perspective, some figures using t-SNE:

in the context of *densely packed continuous parametric curves* and more typical high-dimensional greyscale image data

In all t-SNEs shown in these slides, the *parameters are fixed at the default values*: **perplexity = 30**, **exaggeration = 4**

And a quick comparison with t-SNE…in the context of ***densely packed continuous parametric curves***

Lorenz attractor as example of continuous, densely packed parametric curve in more than 2 dimensions

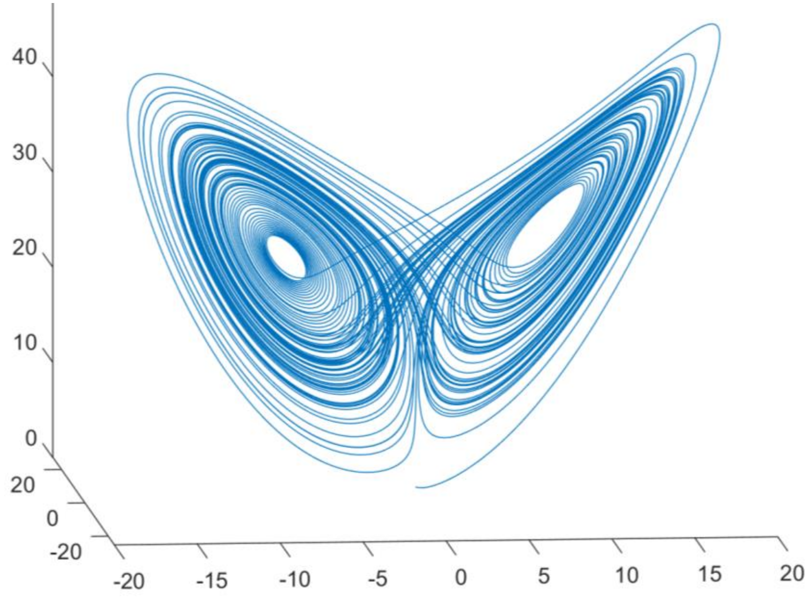

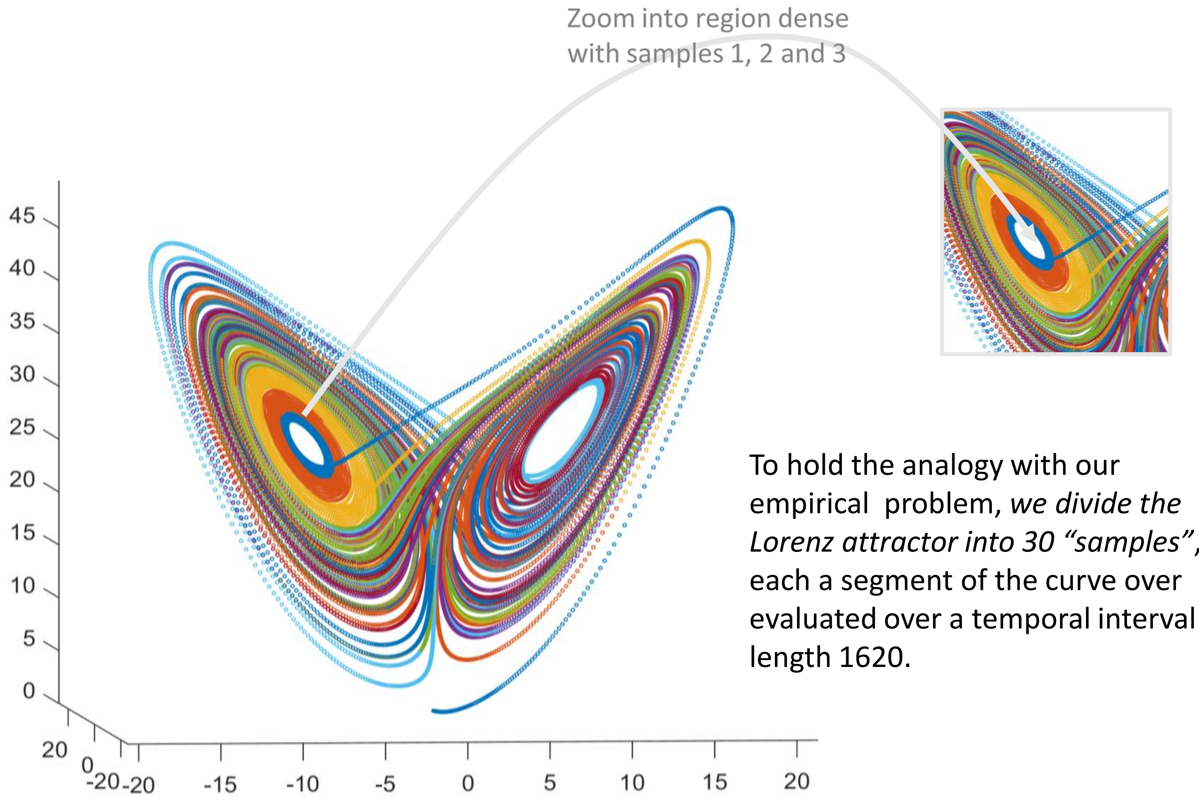

To hold the analogy with our empirical problem, *we divide the Lorenz attractor into 30 "samples"*, each a segment of the curve over evaluated over a temporal interval of length 1620.

20 planar embeddings of the Lorenz attractor using t-SNE with fixed parameters (perplex = 30, exagg = 4). In what is shown below, the order in which attractor points are fed into t-SNE is held constant across the 20 runs.

Twenty t-SNE embeddings of the Lorenz attractor under fixed parameters

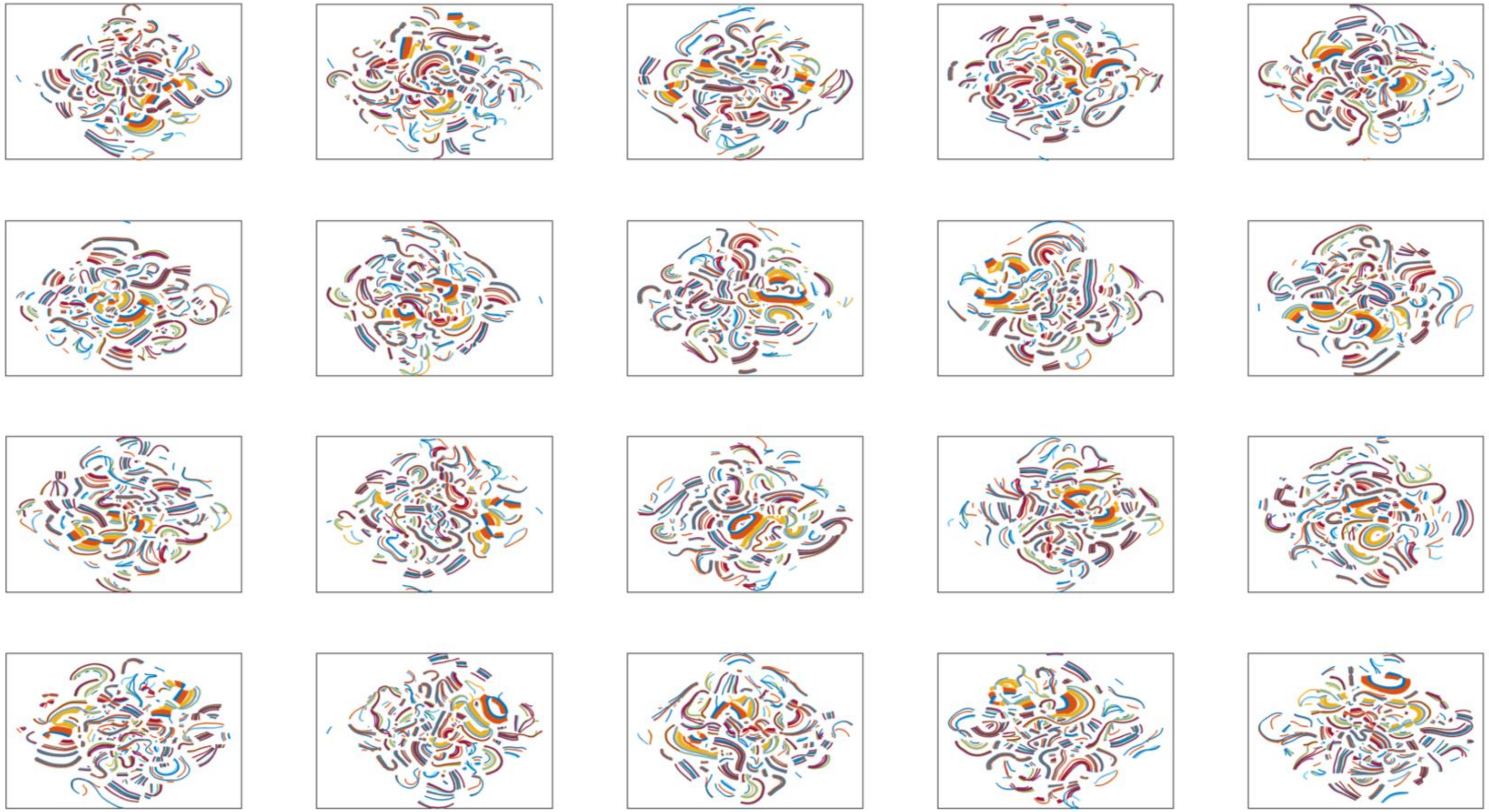

Averages of the embedded points over all runs

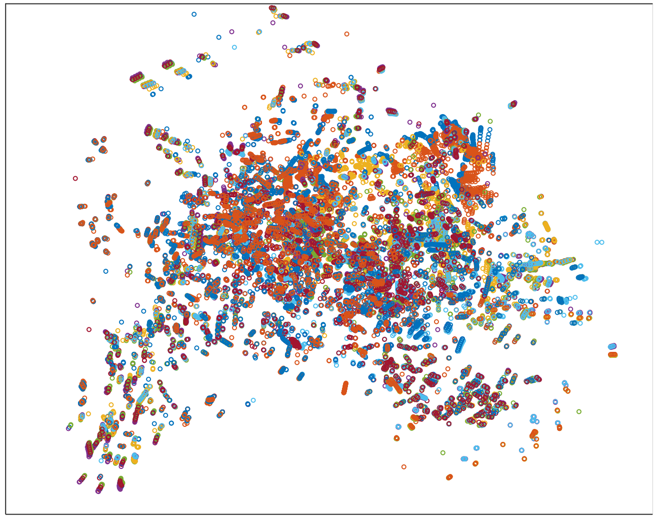

Note that the embeddings does not preserve continuity, density or directional congruence as UMAP did.

Note as well that under fixed parameters and fixed input order, the t-SNE embedding is wildly stochastic

Some figures addressing t-SNE stability…in the less dynamically structured context of grayscale images

Fashion MNIST is a classification benchmarking dataset: 10 classes of clothing presented in 28 x 28 (784 vectorized dimensions) grayscale images

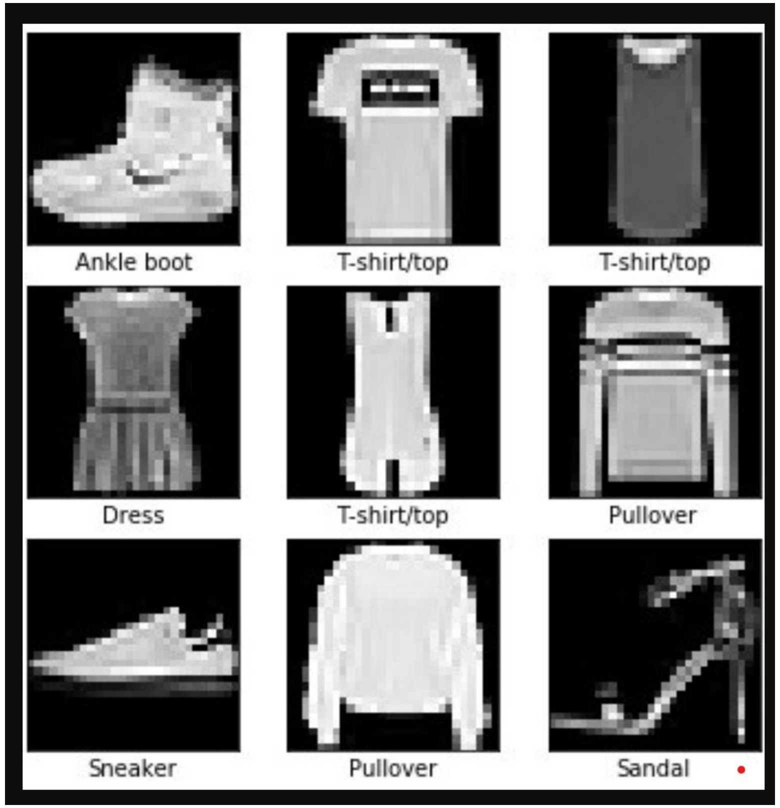

PCA

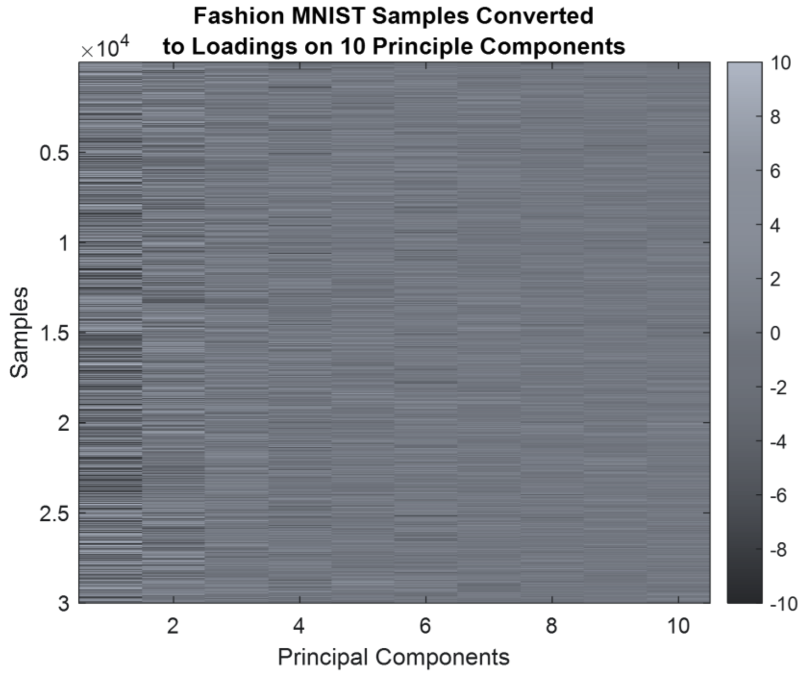

20 planar embeddings of the Fashion MNIST using t-SNE with fixed parameters (perplex = 30, exagg = 4). In what is shown below, the order in which samples are fed into t-SNE is held constant across the 20 runs.

Twenty t-SNE embeddings of Fashion MNIST (10D PCA reduction) under fixed parameters

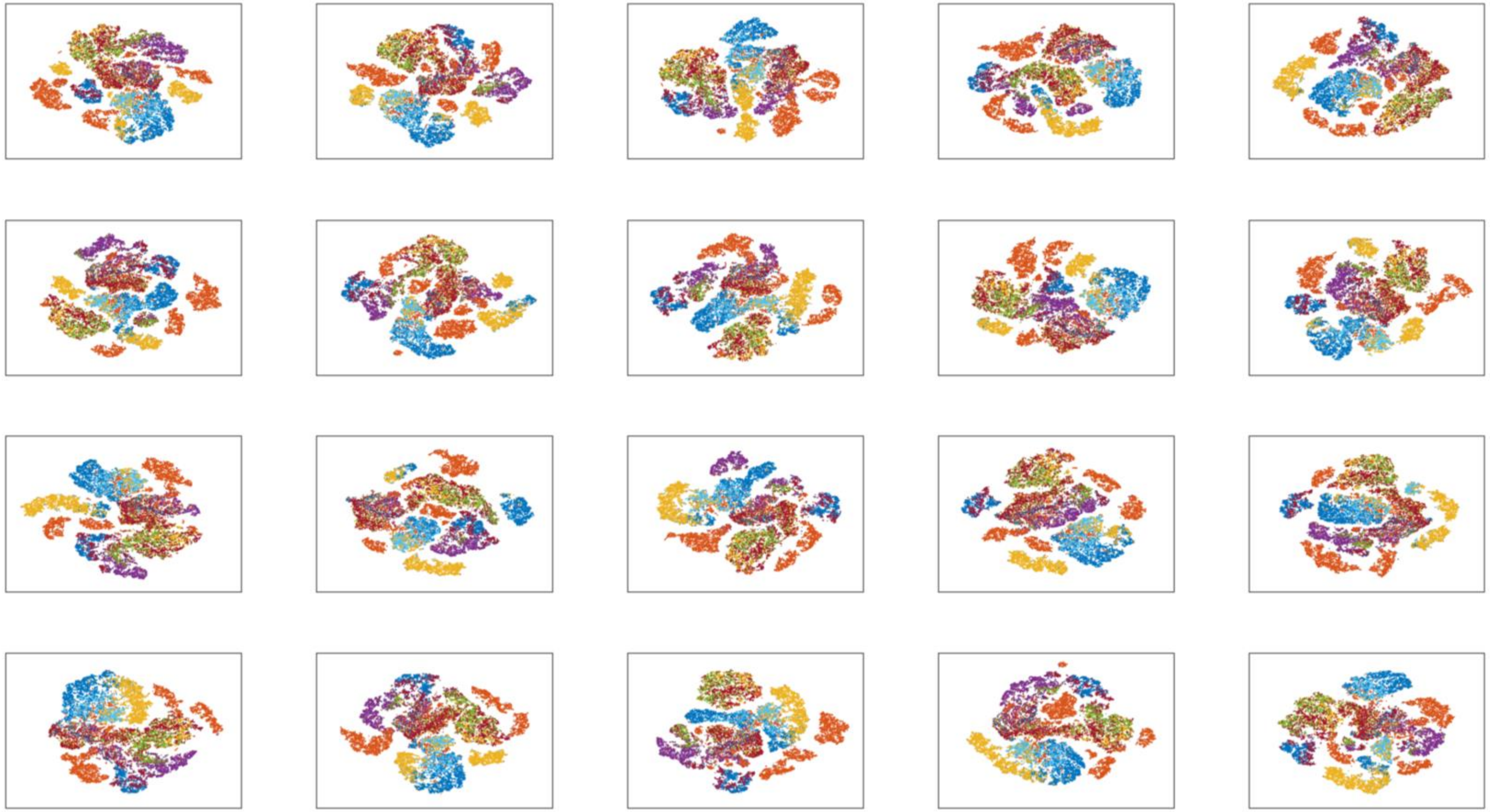

Averages of the embedded points over all runs

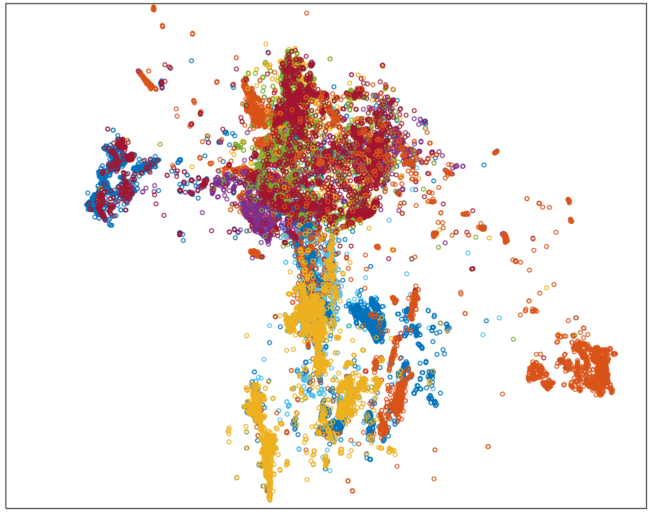

Note that even under fixed parameters and fixed input order, the position of samples in the t-SNE embedding is highly stochastic

There exist less stable parameter regimes for UMAP:

1. in the context of *densely packed continuous parametric curves* and more typical high-dimensional greyscale image data

In all UMAPS shown in these slides, the *parameters are fixed at very small values, shown here to produce unstable embeddings for the two demo datasets*
num_neighbors = 3, **min_dist = 0.1**

Some figures addressing UMAP stability…in the context of ***densely packed continuous parametric curves***

<u>Lorenz attractor</u> as example of continuous, densely packed parametric curve in more than 2 dimensions

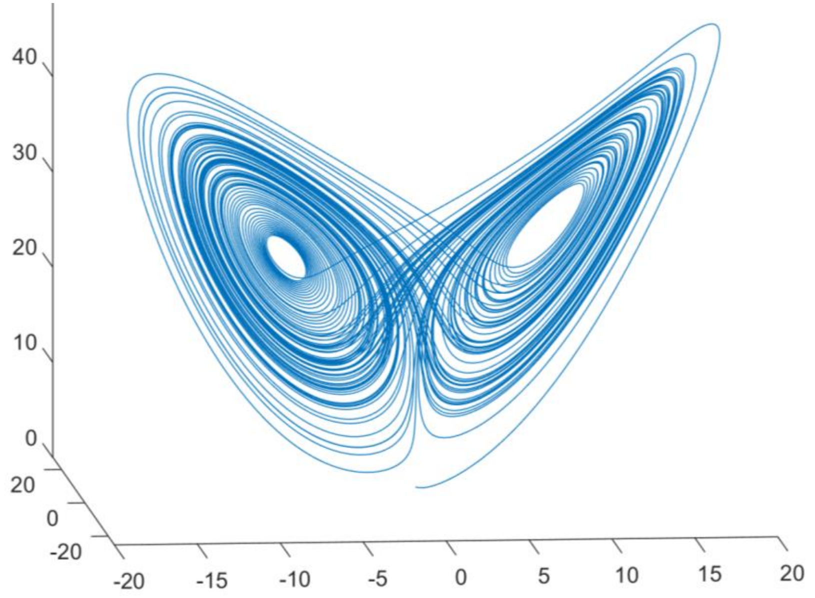

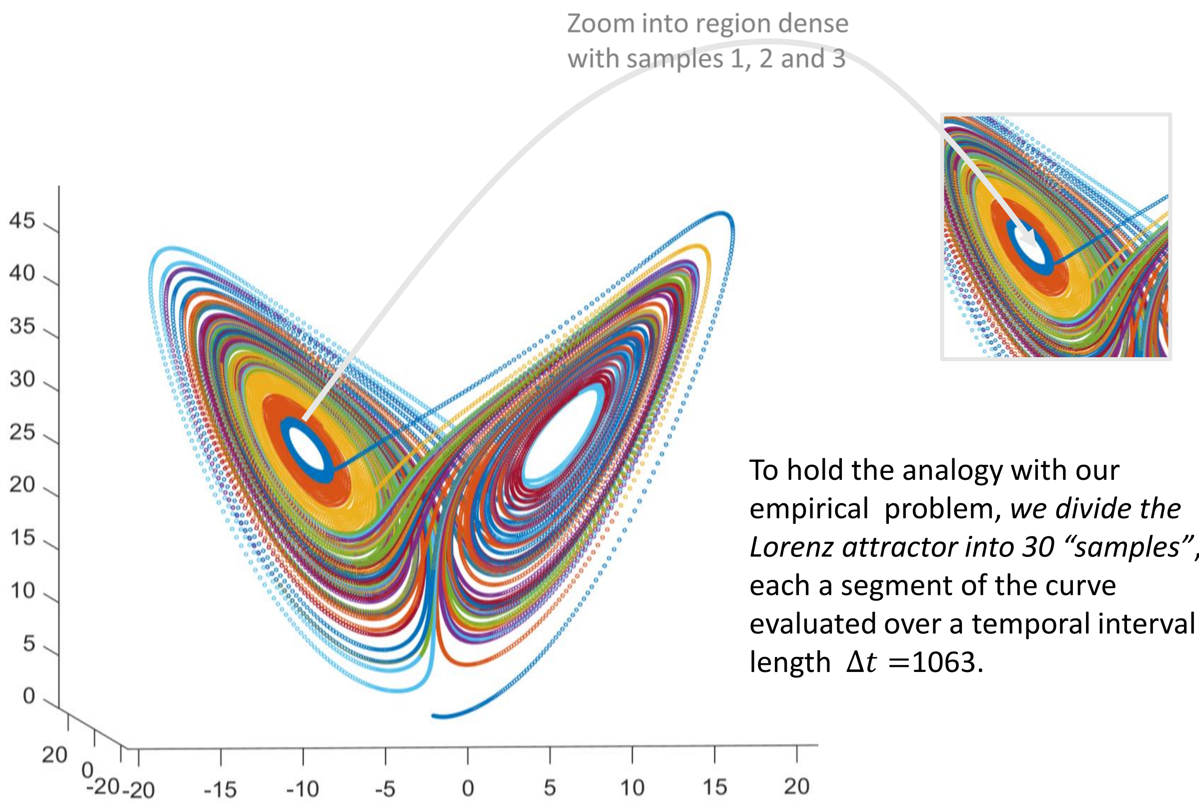

To hold the analogy with our empirical problem, *we divide the Lorenz attractor into 30 "samples"*, each a segment of the curve evaluated over a temporal interval of length Δt =1063.

20 planar embeddings of the Lorenz attractor using UMAP with fixed parameters (nNbrs=3, minDist=0.1). Furthermore, the order in which the attractor points are fed into UMAP is also held constant across the 20 runs, following the actual temporal ordering of the parametric curve.

Twenty UMAP embeddings of the Lorenz attractor under fixed parameters

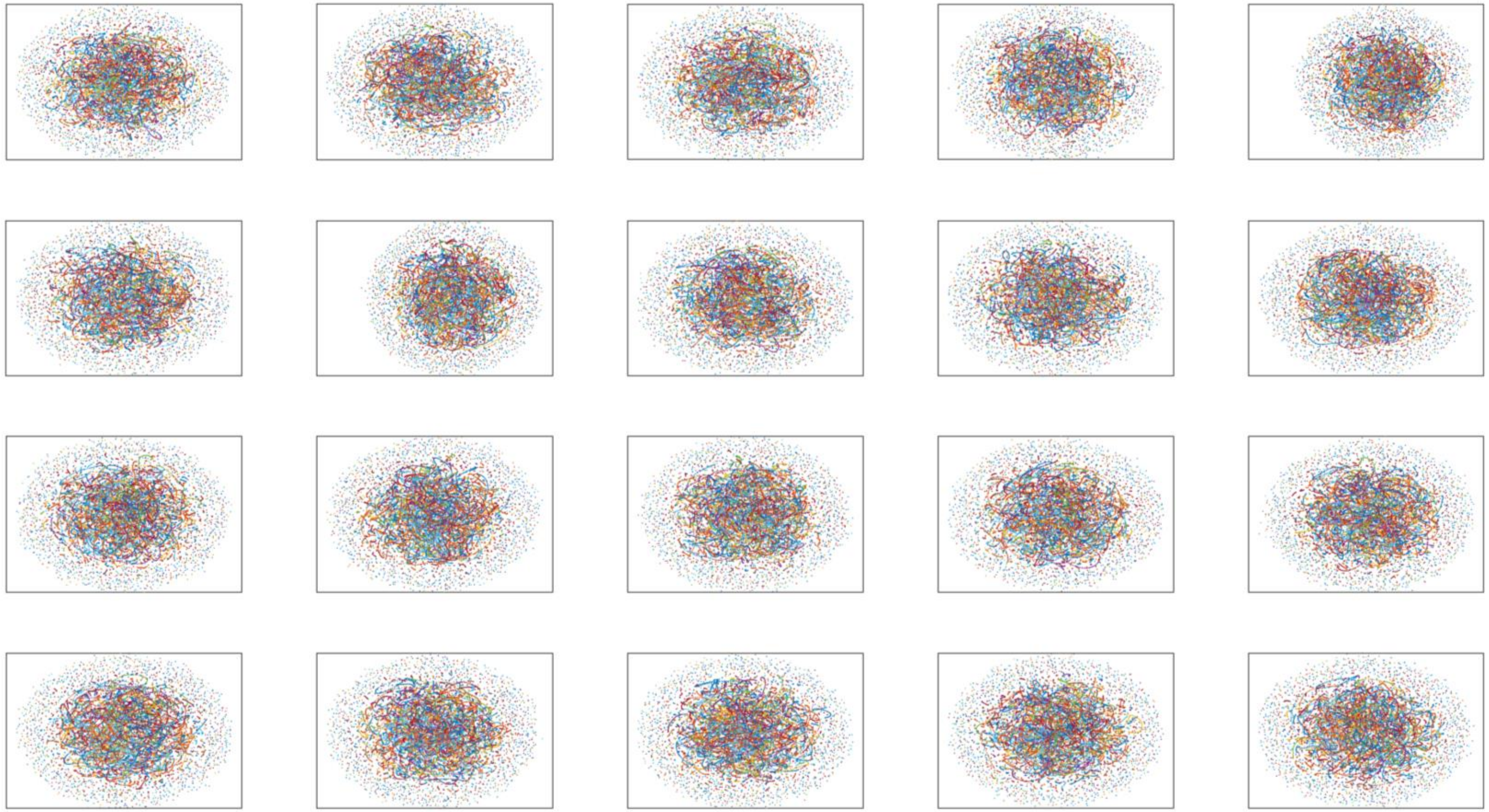

Averages of the embedded points over all runs

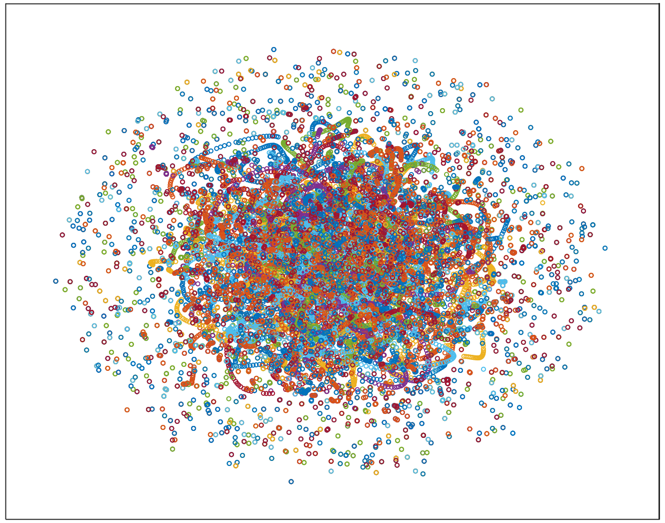

Note that the embedding preserves continuity, also density and directional congruence, e.g. the cluster of samples 1,2 and 3 in the bottom left

Also note that, while not identical, the runs have the kind of aerial view stability that makes averaging points reasonable and doing so yields a sparsified, “cleaned up” version of the individual embeddings that roughly reflects direction and relative density of flow in each embedding, that in turn roughly reflects these features in the high dimensional dynamics

Some figures addressing UMAP stability…in the less dynamically structured context of grayscale images

Fashion MNIST is a classification benchmarking dataset: 10 classes of clothing presented in 28 x 28 (784 vectorized dimensions) grayscale images

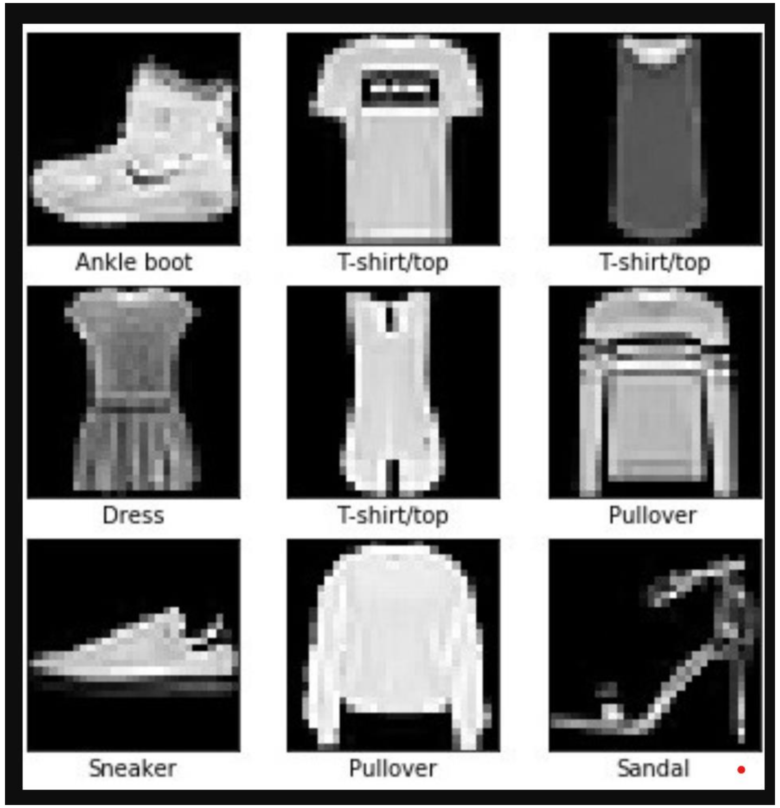

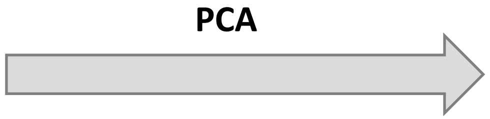

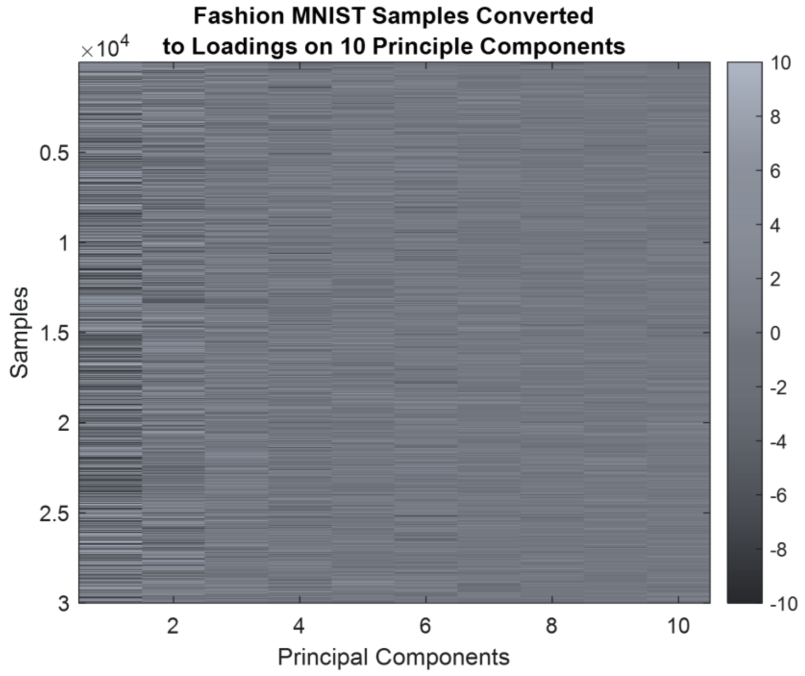

20 planar embeddings of Fashion MNIST using UMAP with fixed parameters (PCA reduced to 10D, nNbrs = 3, minDist = 0.1). The order in which the points are fed into UMAP is held constant across all 20 runs. No specific dynamics or continuity, but a general 10D data embedding problem.

Twenty UMAP embeddings of Fashion MNIST (10D PCA reduction) under fixed parameters

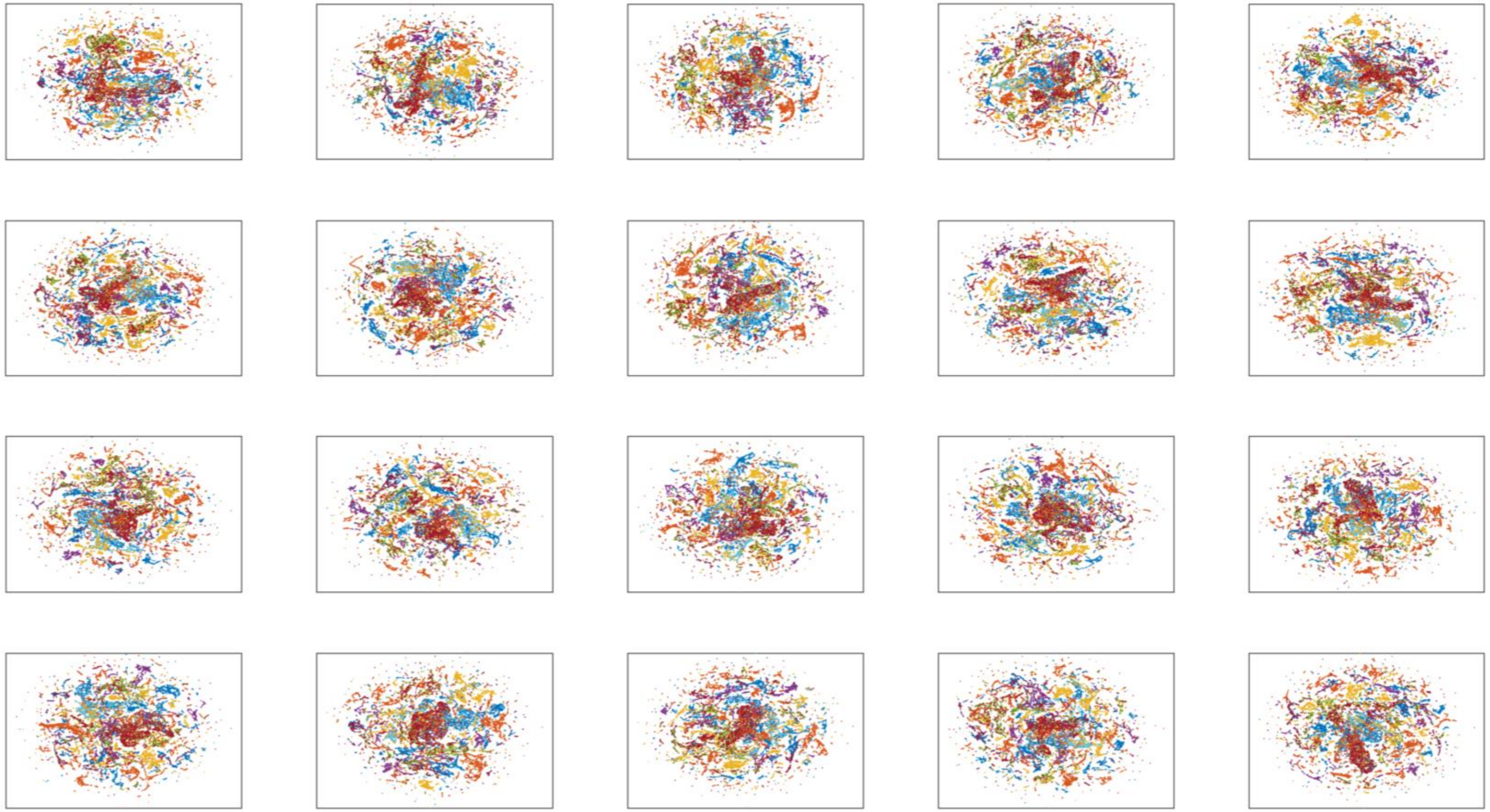

Averages of the embedded points over all runs

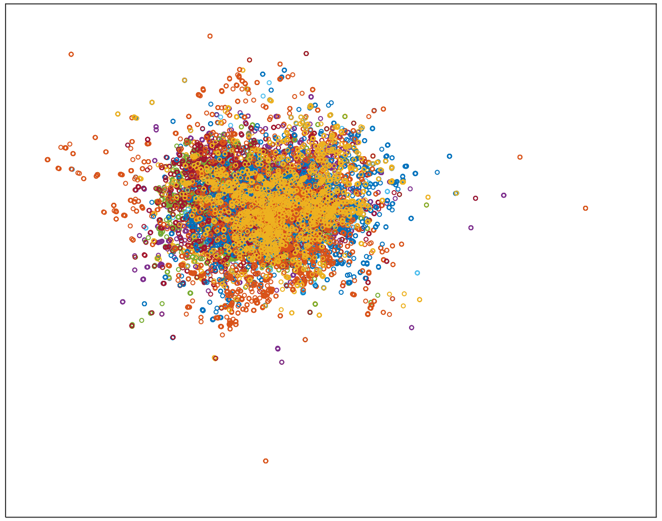

Again note that, while not identical, the runs exhibit a high level of stability that makes averaging points reasonable
