# Supplementary figures and images for: Multiframe Evolving Dynamic Functional Connectivity (EVOdFNC): A Method for Constructing and Investigating Functional Brain Motifs
Source: Front Neurosci. 2022 Apr 19;16:770468. doi: 10.3389/fnins.2022.770468 (PMC9063321; doi:10.3389/fnins.2022.770468)

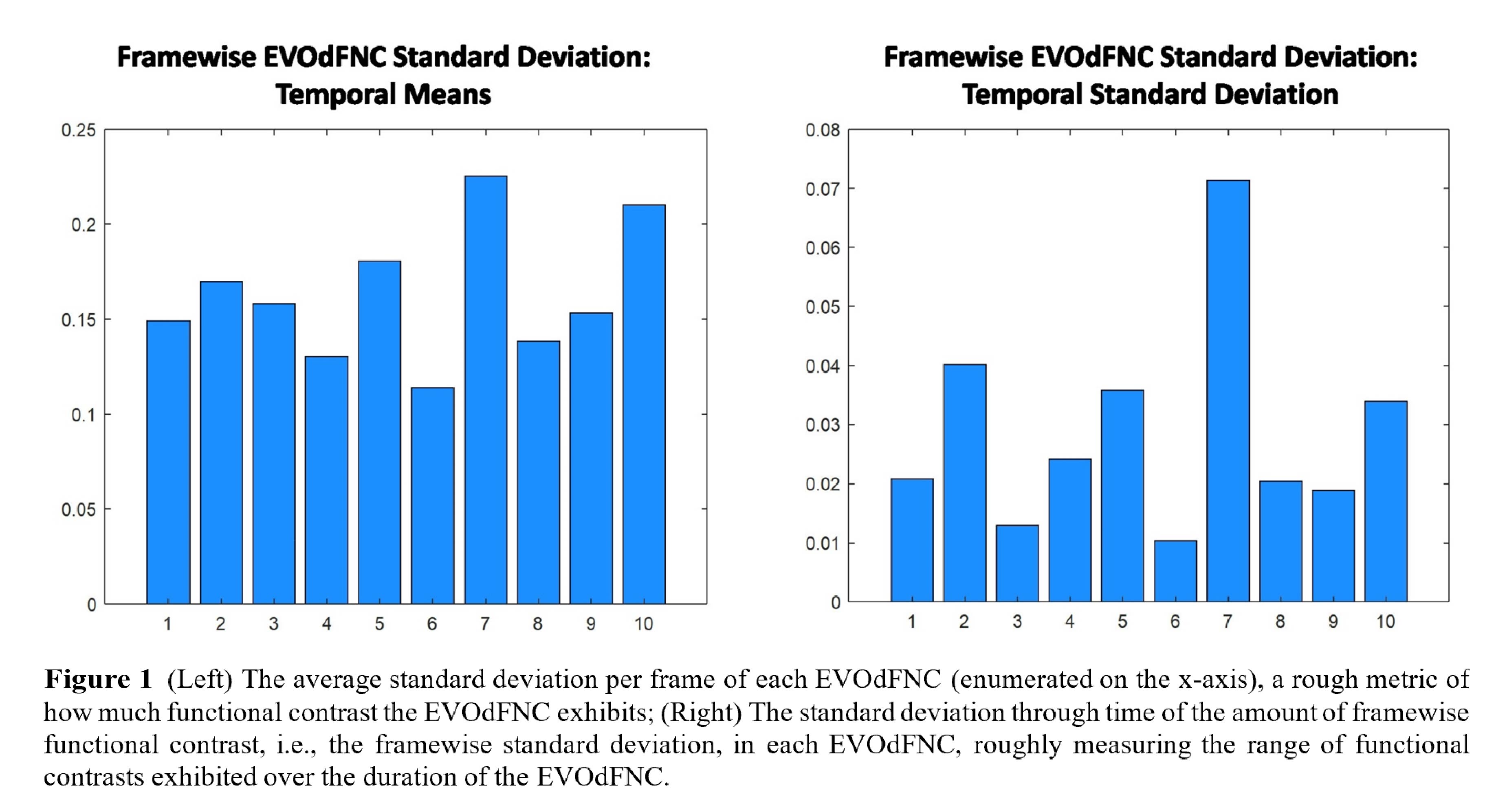

Supplement: Supplementary file 2 [file Image_1.jpg]

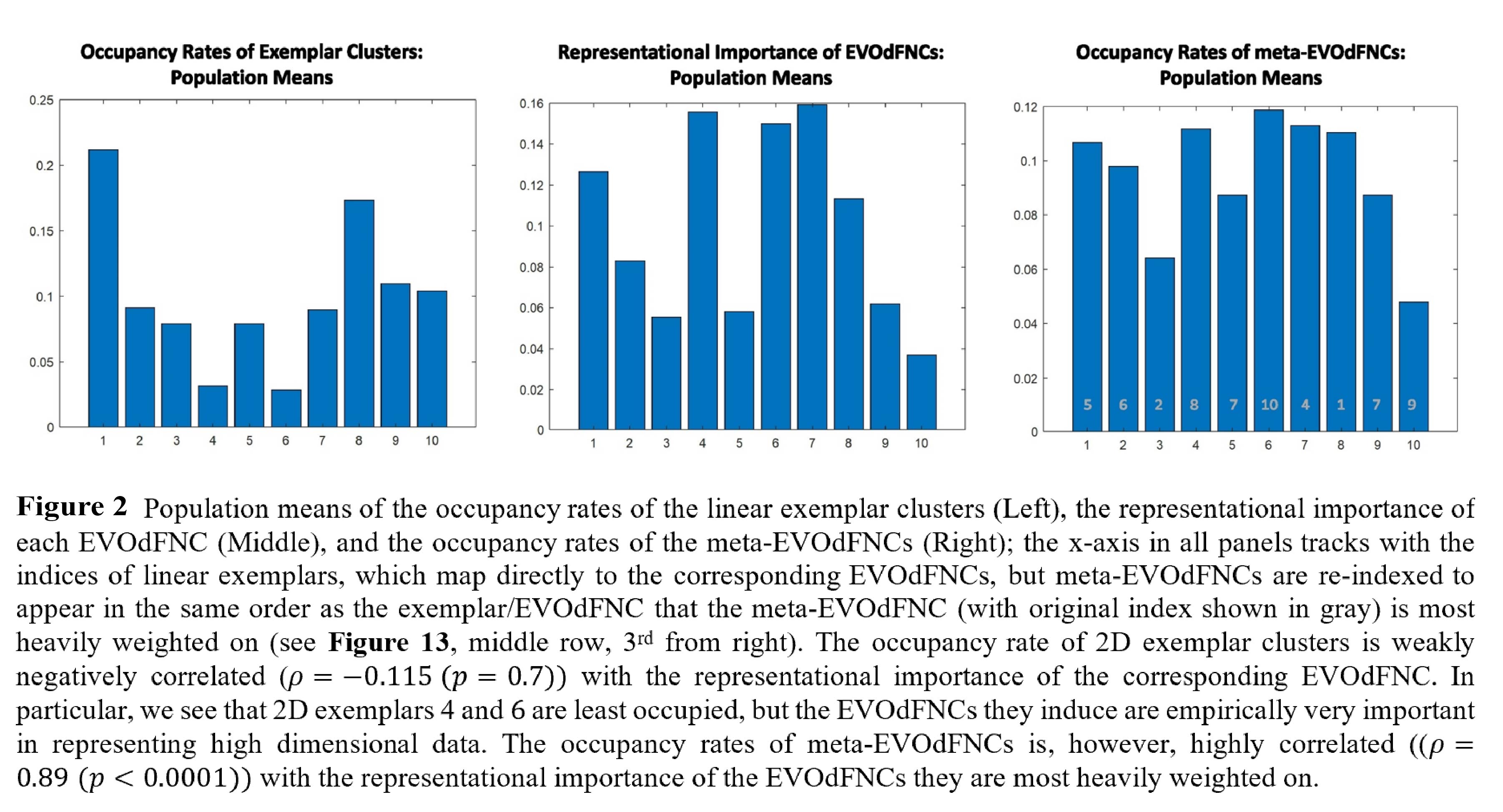

Supplement: Supplementary file 3 [file Image_2.jpg]

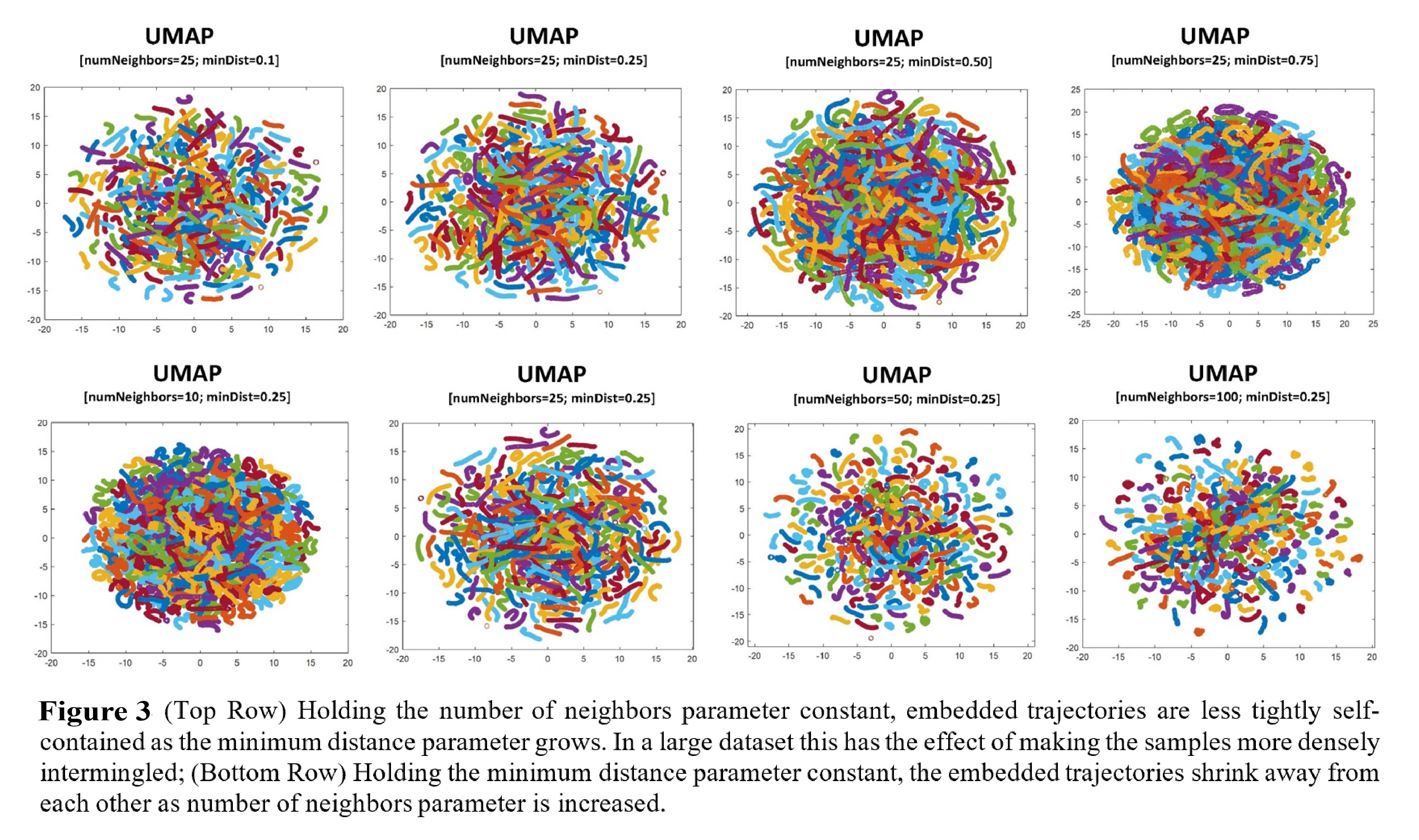

Supplement: Supplementary file 4 [file Image_3.jpg]

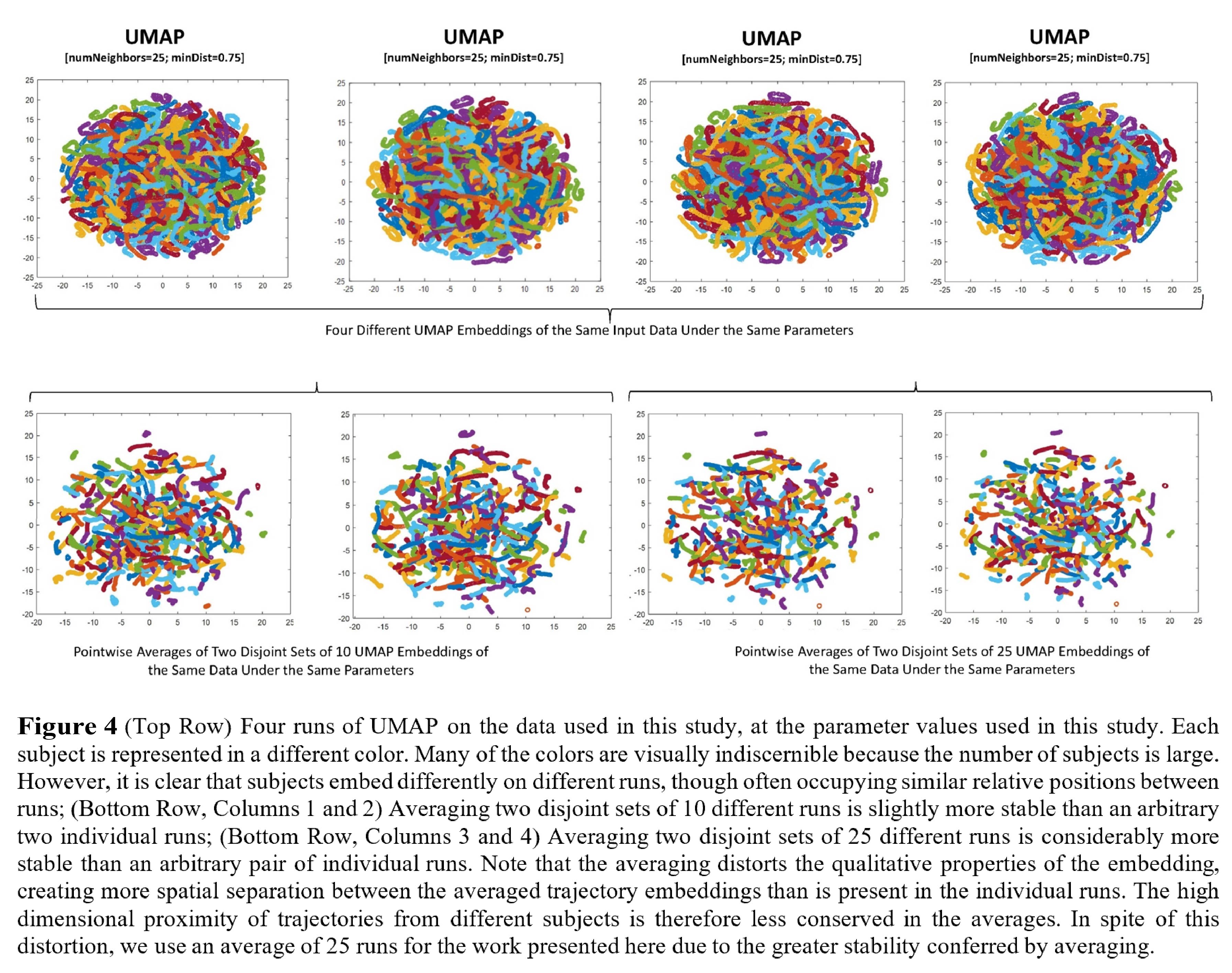

Supplement: Supplementary file 5 [file Image_4.jpg]

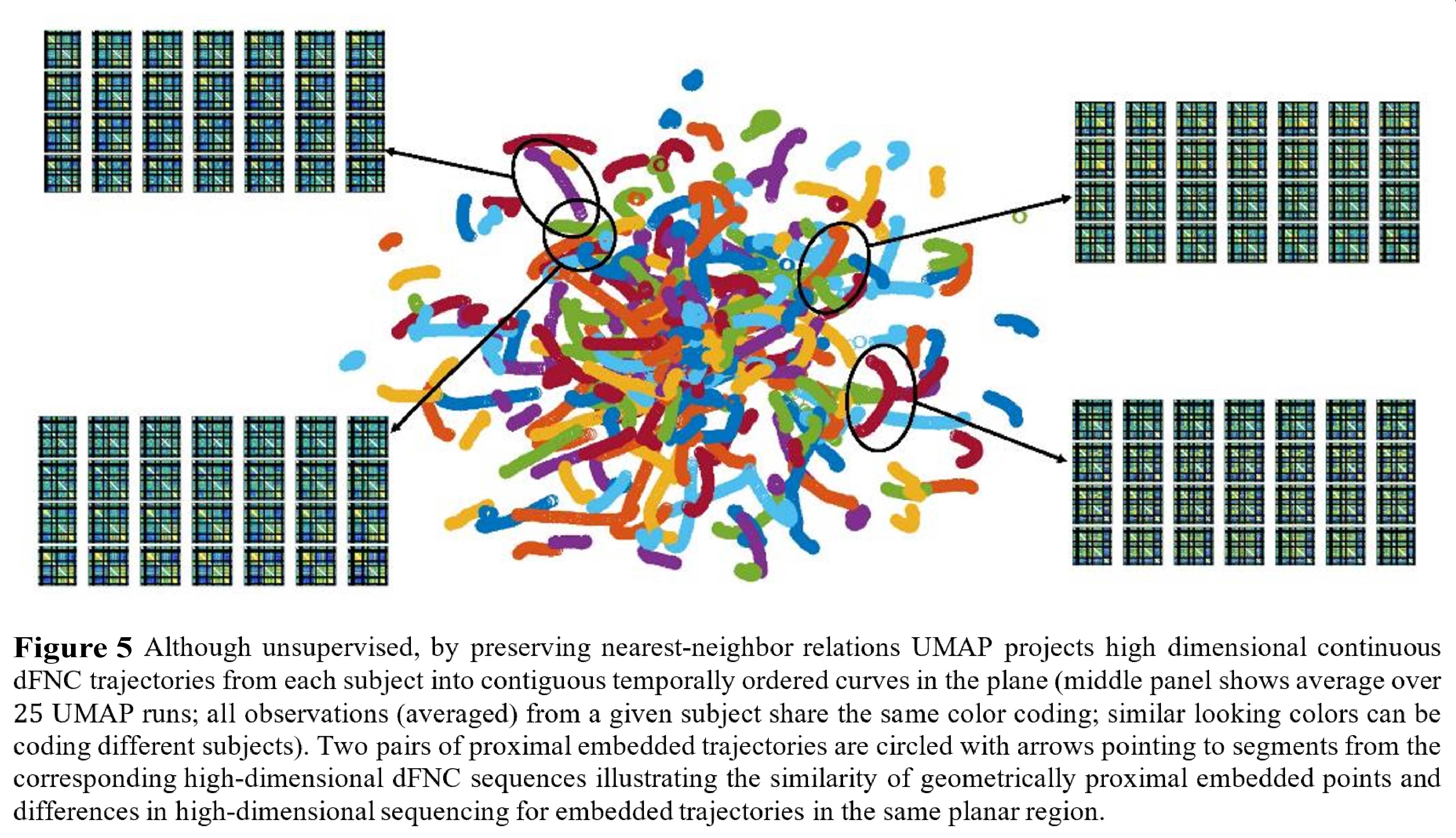

Supplement: Supplementary file 6 [file Image_5.jpg]
